# Exploration of glassy state in Prussian blue analogues

**DOI:** 10.1038/s41467-022-31658-w

**Published:** 2022-07-12

**Authors:** Nattapol Ma, Ryo Ohtani, Hung M. Le, Søren S. Sørensen, Ryuta Ishikawa, Satoshi Kawata, Sareeya Bureekaew, Soracha Kosasang, Yoshiyuki Kawazoe, Koji Ohara, Morten M. Smedskjaer, Satoshi Horike

**Affiliations:** 1 Department of Synthetic Chemistry and Biological Chemistry, Graduate School of Engineering, Kyoto University, Katsura, Nishikyo-ku, Kyoto, 615-8510 Japan; 2 Department of Chemistry, Faculty of Science, Kyushu University, 744 Motooka, Nishi-ku, Fukuoka, 819-0395 Japan; 3 Materials & Devices Laboratory, Institute of Fundamental and Applied Sciences, Duy Tan University, Ho Chi Minh City, 700000 Viet Nam; 4 Department of Chemistry and Bioscience, Aalborg University, Aalborg, 9220 Denmark; 5 Department of Chemistry, Faculty of Science, Fukuoka University, 8-19-1 Nanakuma, Jonan-ku, Fukuoka, 814-0180 Japan; 6 Department of Chemical and Biomolecular Engineering, School of Energy Science and Engineering, Vidyasirimedhi Institute of Science and Technology, Rayong, 21210 Thailand; 7 New Industry Creation Hatchery Center, Tohoku University, Sendai, 980-8579 Japan; 8 Diffraction and Scattering Division, Japan Synchrotron Radiation Research Institute (JASRI), Sayo, Hyogo 679-5198 Japan; 9 AIST-Kyoto University Chemical Energy Materials Open Innovation Laboratory (ChEM-OIL), National Institute of Advanced Industrial Science and Technology (AIST), Yoshida-Honmachi, Sakyo-ku, Kyoto, 606-8501 Japan; 10 Institute for Integrated Cell-Material Sciences, Institute for Advanced Study, Kyoto University, Yoshida-Honmachi, Sakyo-ku, Kyoto, 606-8501 Japan; 11 Department of Materials Science and Engineering, School of Molecular Science and Engineering, Vidyasirimedhi Institute of Science and Technology, Rayong, 21210 Thailand

**Keywords:** Metal-organic frameworks, Coordination chemistry, Solid-state chemistry

## Abstract

Prussian blue analogues (PBAs) are archetypes of microporous coordination polymers/metal–organic frameworks whose versatile composition allows for diverse functionalities. However, developments in PBAs have centred solely on their crystalline state, and the glassy state of PBAs has not been explored. Here we describe the preparation of the glassy state of PBAs via a mechanically induced crystal-to-glass transformation and explore their properties. The preservation of short-range metal–ligand–metal connectivity is confirmed, enabling the framework-based functionality and semiconductivity in the glass. The transformation also generates unconventional CN^−^ vacancies, followed by the reduction of metal sites. This leads to significant porosity enhancement in recrystallised PBA, enabled by further accessibility of isolated micropores. Finally, mechanical stability under stress for successful vitrification is correlated to defect contents and interstitial water. Our results demonstrate how mechanochemistry provides opportunities to explore glassy states of molecular framework materials in which the stable liquid state is absent.

## Abstract (web summary)

Developments in Prussian blue analogues (PBAs) have centred solely on their crystalline state. Here, the authors describe the preparation of the glassy state of PBAs via a mechanically induced crystal-to-glass transformation and explore their properties.

## Introduction

**Fig. 1:**
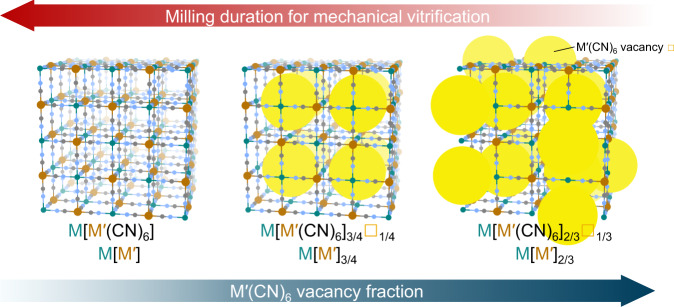
Schematic diagram of the correlation between milling duration for mechanical crystal–glass transformation and vacancy fractions in PBAs with M and M' cations. Green, brown, grey, and blue spheres represent M, M’, C, and N, respectively. H and O atoms are omitted. Yellow spheres represent M'(CN)_6_ vacancies.

Prussian blue analogues (PBAs) are the family of coordination polymers assembled from octahedrally coordinated cyanide bridging metal nodes (M and M') with a parent structure of M[M'(CN)_6_] (Fig. 1)^1^. Variation of metal choices and their oxidation state allows alteration of the vacancies content via the hexacyanometallates withdrawal to compensate for the electroneutrality^2,3^. Prussian blue (PB, Fe^III^[Fe^II^(CN)_6_]_3/4_·*n*H_2_O) itself contains a quarter of the isolated vacancy site^4^. A higher vacancy fraction of one-third fraction can be found in M^II^[M'^III^(CN)_6_]_2/3_·*n*H_2_O, where neighbouring pairs are connected, creating a microporous network structure^2^. With a diverse selection of metal nodes, microporous nature, and unique vacancy/defect arrangement^2^, their contributions cover a wide range of fields from gas^5^ to energy storage^6^, electronic^7^ and ion^8^ transport, magnetic^9^ and optical properties^10^. Since the first synthesis of Prussian blue in 1710^11^, development progress has only revolved around the crystalline phase, either surface modifications^12^ or superstructure formations^13^. PBAs have been long known for their structural diversity, but the amorphous/glassy state has so far been unexplored. Though attempts have been made, neither a stable amorphous state^14,15^ nor a pure phase has been achieved^16^. For example, pressure-induced amorphisation (PIA) of Fe[Co(CN)_6_] to 17.4 GPa does not provide a stable amorphous PBA but decomposes into amorphous carbon nitride, nitrile oxide, metallic iron, and copper instead^14^.

The recent emergence of a new direction toward glass and liquid metal-organic framework/coordination polymers (MOF/CPs) has opened up a new opportunity for discovering unique properties that deviate from the crystalline norm and as a pathway to developing new materials^17–19^. Thus far, only a few CPs and MOFs have shown the capability to undergo the glass transformation process. Most examples are melt-quenched glasses, where the crystal–liquid–glass transition is mainly governed by the counterbalance between entropic/enthalpic driving forces and the kinetics of crystallisation^20^. In contrast, a direct crystal–glass transformation of the mechanical-induced glass has been recognised as another path to prepare the glass and is initiated by plastic deformation under mechanical stress^21,22^. However, the factors that dictate the mechanical-induced phase transition have not yet been established. Limited 3D MOF/CP glasses libraries also restrict a rich discussion on the correlation between glassy behaviour and intrinsic defect/vacancy or structural variation. Tuneable vacancy content, pore structure, and compositional versatility make classical PBAs a suitable representative compound to enable a systematic study and precise control of functionality in MOF/CP glasses.

In this study, we report the discovery of the glassy state in PBAs through mechanical-induced crystal–glass transformation. The glassy nature was confirmed by X-ray diffuse scattering pattern, preservation of short-range order through pair distribution function (PDF) analysis, and the presence of glass transition behaviour. Unlike other CP glasses, alteration of the oxidation state while generating CN^−^ vacancies was observed via ^57^Fe Mössbauer spectroscopy and elemental analysis. We observed an anomalous gas adsorption enhancement in recrystallised PBA with semiconductivity retention in PBA glass. Finally, we identify the origin of crystal–glass transformation by DFT under mechanical stress and generalise the correlation between the mechanical properties and glass transition difficulties by comparing the contribution of the presence of hexacyanometallate vacancies and the effect of the guest molecules.

## Results and discussion

### Crystal–glass transformation

Representative PBA containing 1/3 hexacyanoferrate vacancy, K_2*x*/3_Cu^II^[Fe^II^_*x*_Fe^III^_*1−x*_(CN)_6_]_2/3_□_1/3_·*n*H_2_O (Cu[Fe]_2/3_), was synthesised via an aqueous precipitation method with a composition of K_0.32_Cu[Fe(CN)_6_]_0.63_·*n*H_2_O (Table 1, Supplementary Tables 1 and 2)^5,6^. Powder X-ray diffraction (PXRD) of the Cu[Fe]_2/3_ resembles the face-centred cubic diffraction pattern of Prussian blue, Fe^III^[Fe^II^(CN)_6_]_3/4_□_1/4_·*n*H_2_O (Fe[Fe]_3/4_, *a* = 10.166 Å), with Bragg peaks shifting toward a higher 2*θ* (Supplementary Fig. 1)^4^. Unlike Fe[Fe]_3/4_, Rietveld refinement of synchrotron PXRD data (Supplementary Fig. 2, λ = 0.2020 Å) revealed that Cu[Fe]_2/3_ contains a slightly smaller cell parameter of 10.067 Å (*Fm*−3*m*, *a* = 10.067 Å, *R*_p_ = 4.14%, *R*_wp_ = 5.87%). Thermogravimetric analysis (TGA, Supplementary Fig. 3) of Cu[Fe]_2/3_ shows two steps of weight loss associated with the release of interstitial and coordinated water with a total loss of ca. 26 wt% (*n* = 4).

**Table 1:** Summary of crystalline and glass samples.

Starting sample	Abbreviation	Note
K_2*x*/3_Cu^II^[Fe^II^_*x*_Fe^III^_*1−x*_(CN)_6_]_2/3_□_1/3_·*n*H_2_O	Cu[Fe]_2/3_	
(K_0.32_Cu[Fe(CN)_6_]_0.63_·*n*H_2_O)	Cu[Fe]_2/3_-g	Glassy state, 2 h milling
	Cu[Fe]_2/3_'	Dehydrated Cu[Fe]_2/3_
	Cu[Fe]_2/3_'-g	Glassy state, 2 h milling of Cu[Fe]_2/3_'
	Cu[Fe]_2/3_-c	Recrystallised state: expose Cu[Fe]_2/3_-g to 85 RH% vapour at 80 °C for 72 h
	Cu[Fe]_2/3_-c'	Dehydrated Cu[Fe]_2/3_-c
	Cu[Fe]_2/3_-h	Expose Cu[Fe]_2/3_ to 85 RH% vapour at 80 °C for 72 h
Fe^III^[Fe^II^(CN)_6_]_3/4_□_1/4_·*n*H_2_O	Fe[Fe]_3/4_	
	Fe[Fe]_3/4_-g	Glassy state, 144 h milling
	Fe[Fe]_3/4_-c	Expose Fe[Fe]_2/3_-g to 85 RH% vapour at 80 °C for 72 h

**Fig. 2:**
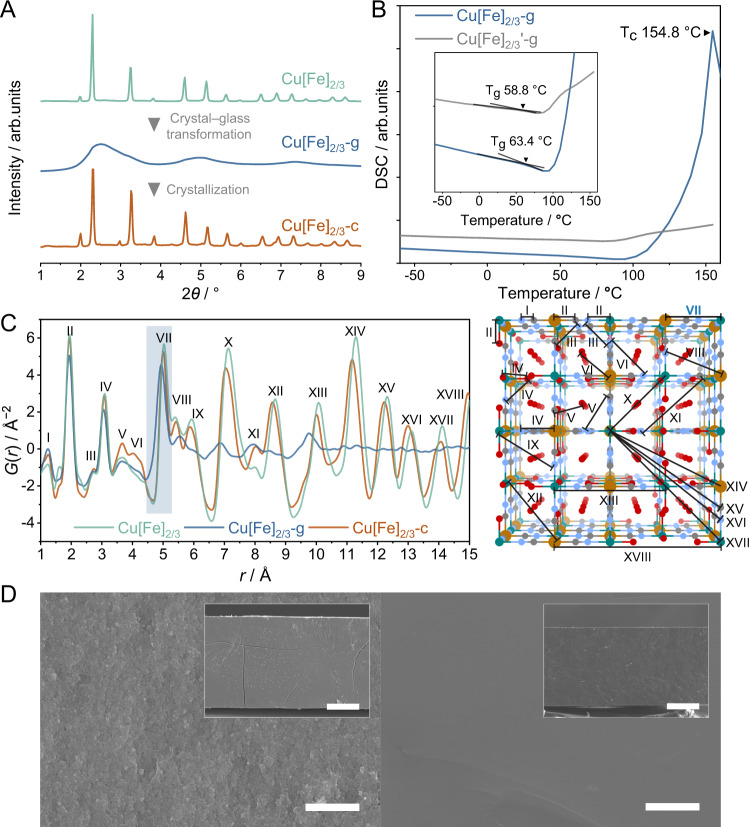
Crystal–glass transformation and recrystallisation of Cu[Fe]_2/3_. **A** Synchrotron PXRD (λ = 0.2020 Å) of Cu[Fe]_2/3_ (green), Cu[Fe]_2/3_-g (blue), and Cu[Fe]_2/3_-c (brown). **B** Enthalpic response comparison between Cu[Fe]_2/3_-g (blue) and Cu[Fe]_2/3_'-g (grey) at 30 °C min^−1^. **C** Pair distribution function (PDF) and PDF peak assignment of Cu[Fe]_2/3_ (green), Cu[Fe]_2/3_-g (blue), and Cu[Fe]_2/3_-c (brown). The blue highlight presents Cu–Fe correlation pair (label VII). The selected peak assignments are highlighted in the schematic diagram of the crystalline Cu[Fe]_2/3_. Cu, Fe, C, N, and O atoms are represented as green, brown, grey, blue, and red spheres, respectively. H atoms are omitted for clarity. **D** Comparison of cross-sectional SEM images of hot-pressed Cu[Fe]_2/3_ (left) and Cu[Fe]_2/3_-g (right). Scale bar = 2 μm and 100 μm for enlarging and inset, respectively. The definition of the samples’ abbreviations can be found in Table 1. Source data are provided as a Source Data file.

To obtain the glassy state of Cu[Fe]_2/3_, we conducted mechanical milling treatment of crystalline Cu[Fe]_2/3_ under Ar atmosphere with a milling time of 120 min. The conditions are identical to the formation of glassy state Cd(1,2,4-triazole)_2_(H_2_PO_4_)_2_ reported in our previous study^22^. Throughout the milling process, the temperature remains below 34 °C by directly monitoring the temperature inside the jar (Supplementary Fig. 4). After ball milling, the colour of Cu[Fe]_2/3_ changes from dark green to dark blue (Supplementary Figs. 5 and 6). Synchrotron PXRD of the Cu[Fe]_2/3_ glass (Cu[Fe]_2/3_-g, Fig. 2A) demonstrates a broad diffuse scattering, proposing an amorphous nature^21,22^. Though the Cu[Fe]_2/3_-g retains two steps of weight loss in TGA (Supplementary Fig. 3), the second step is much more gradual. The thermal behaviour of Cu[Fe]_2/3_-g was further studied by a closed system differential scanning calorimetry (DSC) analysis. Cu[Fe]_2/3_-g exhibits a based-line shifting at the glass transition temperature (*T*_*g*_) of 44.7 to 63.4 °C at a heating rate of 10 to 30 °C min^−1^, respectively (Fig. 2B and Supplementary Fig. 7)^21,23^. As heating continues, an exothermic recrystallisation peak is observed at the crystallisation temperature (*T*_c_) of 144.8 and 154.8 °C at 10 and 30 °C min^−1^, respectively. After the DSC measurement, PXRD of the sample shows partial restoration of Bragg diffraction, confirming the recrystallisation process (Supplementary Fig. 8). In contrast, heating in an open system (Ar flow) does not provide recrystallisation, as confirmed in the variable temperature PXRD data (Supplementary Fig. 9). To clarify the dissimilarity between the closed and open systems in DSC, we prepared an activated Cu[Fe]_2/3_ at 95 °C under vacuum (Cu[Fe]_2/3_')^5^. Although the PXRD remains unchanged (Supplementary Fig. 10), total removal of interstitial water (ca. 8.2 wt%) was observed in TGA (Supplementary Fig. 11). Mechanical induced crystal–glass transformation is also applicable to Cu[Fe]_2/3_' yielding Cu[Fe]_2/3_'-g with a diffuse scattering (Supplementary Fig. 10), a *T*_g_ of 58.8 °C (30 °C min^−1^, Fig. 2B), and a negligible weight loss at the *T*_g_ (<0.2%, Supplementary Fig. 11). DSC measurements of Cu[Fe]_2/3_'-g in a closed system did not provide any exothermic peak, suggesting that water vapour generated from the interstitial water in Cu[Fe]_2/3_-g is required to aid the recrystallisation process due to its porous nature^24^. It is worth mentioning that thermal annealing alone is sufficient for dense CP glasses^21,22^.

Encouraged by the DSC results and vapour assisted recrystallisation in some MOF/CP glasses, we attempted to reconstruct the crystalline framework from the glassy state^17,21,24^. The Cu[Fe]_2/3_-g was exposed to humid air (85 RH%) at 80 °C for 72 h. Again, the colour alters to brown (Supplementary Fig. 5). The synchrotron PXRD pattern confirms recrystallisation with Bragg peaks mostly resembling the Cu[Fe]_2/3_ and slightly shifting to the higher 2*θ* value, suggesting a shorter cell length (Fig. 2A and Supplementary Fig. 12). Additional defects potentially cause symmetry lowering in Cu[Fe]_2/3_-c^25^. The recrystallised state is henceforth denoted as Cu[Fe]_2/3_-c. TGA reveals that the interstitial water content is boosted to ca. 27.5 wt% (Supplementary Fig. 13). Activation of Cu[Fe]_2/3_-c provides a highly porous Cu[Fe]_2/3_-c'. Recrystallisation was also tracked by scanning electron microscopy (SEM). After the recrystallisation process, recovery of discrete crystal nature was observed with an indistinguishable domain size as the Cu[Fe]_2/3_ (Supplementary Fig. 14).

### Chemical structures in glass and recrystallised phases

With an absence of Bragg diffraction observed in the glassy state, we further probed the short–to–intermediate-range order in the local structure of the Cu[Fe]_2/3_-g via synchrotron X-ray total scattering. Fourier transformation and Lorch modification of the Faber–Ziman total structure factor, *S*(*Q*), to pair distribution functions (PDFs), *G*(*r*), provide information on atom pair distance histograms (Fig. 2C)^26^. Peak features of the glass phase remain mostly identical to the crystalline Cu[Fe]_2/3_ up to ca. 5.3 Å with slightly reduced amplitude. This corresponds to the retention of the C≡N, Fe–C, and Cu–N bonds of the coordination network. Besides, the Cu–O bond represents the coordinated water adjacent to the hexacyanoferrate vacancy site. The preserved range includes the nearby Cu–Fe correlation pair (4.94 Å, label VII in Fig. 2C) and the preservation of ν(C≡N) band between 2177 and 2104 cm^−1^ under infrared spectroscopy (Supplementary Fig. 15), suggesting that metal–ligand–metal (Cu–N≡C–Fe) connectivity remains intact after the mechanical-induced crystal–glass transformation process. PDF of Cu[Fe]_2/3_-g suggests a slightly smaller pair distance as compared to the Cu[Fe]_2/3_^17,20,22^. Note, the Cu–Fe correlation of Cu[Fe]_2/3_ is ca. 5.03 Å, corresponding to the cell parameter of 10.067 Å as suggested by Rietveld refinement. Above 5.3 Å, the PDF of the Cu[Fe]_2/3_-g exhibits lower peak intensity and peaks broadening as the periodicity diminishes. Partial retention of correlations between the diagonal Cu–Cu/Fe–Fe (X), diagonal Cu–Fe (XII), and in-plane Cu–Cu/Fe–Fe (XIII) nearest neighbours in Cu[Fe]_2/3_-g is observed at ca. 6.86, 7.94, and 9.76 Å, respectively. These values are smaller than the pair distances of ca. 7.13, 8.66, and 10.06 Å observed in Cu[Fe]_2/3_ (Fig. 2C and Supplementary Fig. 16). Apart from the slight deviation of octahedral geometry around Cu and Fe with bond angles expected to alter from 90° in Cu[Fe]_2/3_-g, shortened pair distance occurs due to the partial reduction of Fe^III^ to Fe^II^, which will be discussed in the next section^27^.

After the recrystallisation, it shows the restoration of pair correlation above 5.3 Å in the Cu[Fe]_2/3_-c (Fig. 2C). Uniform peaks shifting toward a lower pair distance confirm the lattice parameter shrinkage due to the partial reduction of Fe centres with a Cu–Fe correlation of 5.00 Å. The PDF features of Cu[Fe]_2/3_-c are identical to the Cu[Fe]_2/3_ peaks without any additional phase (Supplementary Figs. 16 and 17). Though most of the features are restored, the PDF contains slightly diminished peak amplitudes, suggesting the existence of partial disorder^28^. The inclusion of additional interstitial water in Cu[Fe]_2/3_-c, suggested by TGA (Supplementary Fig. 13), also results in the amplification of pair correlation at ca. 3.7 Å (V) and 4.0 Å (VI). Note that the positions also contain other pair correlations, including C–N, C–C, and N–N.

Another important and preferable characteristic of glass is its ability to form a grain-boundary-free monolith^19,21^. We first prepared a thin monolith disc of Cu[Fe]_2/3_-g with a thickness of ca. 250 µm and a diameter of 16 mm (Supplementary Fig. 18) via the hot-pressing technique at 80 °C under vacuum. Cross-section SEM of Cu[Fe]_2/3_-g monolith disc reveals a grain-boundary-free character of glass, whereas Cu[Fe]_2/3_ retains its microscopic crystalline morphologies with noticeable cracks under identical conditions (Fig. 2D).

### Alteration of oxidation states

Oxidation states of the M'–CN terminal are distinguishable through the ν(C≡N) shifts between 2000 and 2200 cm^−1^ via infrared spectroscopy^29^. The CN^−^ linker binds the metal nodes via a *σ*-donation of a weak antibonding 5*σ* orbital together with a metal *π*-back-donation to antibonding *π*-orbital. A decrease in ν(C≡N) mode is expected when a transition metal node with lower oxidation is presented in the structure due to the addition of electrons in the 2pπ* antibonding orbital. In Cu[Fe]_2/3_ (Supplementary Fig. 15), we found coexistence of ν(C≡N) band at 2177 and 2104 cm^−1^ corresponding to Fe^III^–C≡N–Cu^II^ and Fe^II^–C≡N–Cu^II^, respectively. The presence of mixed valency corresponds to the incorporation of A-site K^+^ cation^29,30^. Cu[Fe]_2/3_-g, Cu[Fe]_2/3_-c, and Cu[Fe]_2/3_-c', on the other hand, exhibit domination of single ν(C≡N) mode at 2087–2100 cm^−1^ from Fe^II^–C≡N–Cu^II^, suggesting that partial reduction of Fe^3+^ nodes occurs during ball milling process^31^.

We performed ^57^Fe Mössbauer spectroscopy at room temperature to support these oxidation state assignments (Supplementary Fig. 19). The fitting parameters are summarised in Supplementary Table 2. Best-fitting for the Cu[Fe]_2/3_ requires two doublets with isomer shift (IS) and quadrupole splitting (QS) of −0.088 mm s^−1^, 0.179 mm s^−1^ and −0.159 mm s^−1^, 0.556 mm s^−1^, confirming the coexistence of hexacyanoferrate with 51.1% Fe^II^ (S = 0) and 48.9% Fe^III^ (S = 1/2), respectively^32^. After transformation to Cu[Fe]_2/3_-g, we observed a significant shifting in the Fe^II^ (S = 0) and Fe^III^ (S = 1/2) ratio to 67.0% and 33.0%, respectively. This Fe^II^ (S = 0) dominancy remains unchanged for recrystallised Cu[Fe]_2/3_-c and Cu[Fe]_2/3_-c'. Indeed, these assignments are also close parallel to the Fourier transform infrared data, confirming that the Fe^III^ site is partially reduced to Fe^II^ via the mechanical milling process^31^.

Nevertheless, to satisfy the electroneutrality principle, partial elimination of CN^−^ is required. To confirm, we further tracked the change in weight fraction of C and N via elemental analysis (Supplementary Table 3). After 2 h of mechanical milling, C and N weight fractions reduced from 23.7% (standard deviation, SD = 0.34) and 28.4% (SD = 0.77) to 22.7% (SD = 0.20) and 27.0% (SD = 0.56), respectively, confirming the formation of CN^−^ vacancies to accommodate the reduction from Fe^III^ to Fe^II^^33^. We hypothesise that CN^−^ is released during the milling process as HCN gas. Raman spectroscopy also shows a higher degree of deformation around N–Cu–N and Fe–CN in Cu[Fe]_2/3_-g, which is also retained in Cu[Fe]_2/3_-c and Cu[Fe]_2/3_-c' (Supplementary Fig. 20)^34,35^. Moreover, Fe K-edge extended X-ray absorption fine structure (EXAFS) fittings reveal the change in the coordination environment around the Fe centre (Fe–CN) after the mechanical milling process, where the coordination number decreases from 6.0 in Cu[Fe]_2/3_ to 4.4 in Cu[Fe]_2/3_-g (Supplementary Figs. 21 and 22, and Supplementary Table 4)^33,36,37^. Though the reassociation of partially dissociated Fe–CN is observed in Cu[Fe]_2/3_-c after recrystallisation, the coordination number of 5.4 determined by EXAFS supports the formation of the unsaturated metal site due to the partial elimination of CN^−^.

### Enhanced porosity via crystal–glass–crystal transformation

**Fig. 3:**
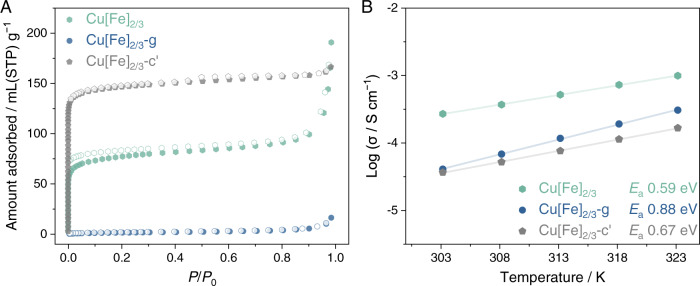
N_2_ adsorption and electronic conductivity. **A** N_2_ adsorption isotherms at 77 K of Cu[Fe]_2/3_ (green), Cu[Fe]_2/3_-g (blue), and Cu[Fe]_2/3_-c' (grey). Adsorption and desorption processes are represented by filled and hollow symbols, respectively. **B** Electronic conductivities of Cu[Fe]_2/3_, Cu[Fe]_2/3_-g, and Cu[Fe]_2/3_-c' measured in an Ar atmosphere. Source data are provided as a Source Data file.

The existence and nature of the extended micropore networks in PBAs rely on the long-range arrangement of correlated hexacyanometallate vacancies^2,5^, implying the presence of inaccessible cavities where the gas molecules cannot diffuse in during the experimental timescale^38^. Partial amorphisation of some crystalline MOFs through a lightly shear/uniaxial deformation has been demonstrated to open up these closed pockets through the formation of dangling linkers in computational studies. Up to 48.5% and 20.8% increases in the accessible surface area have been demonstrated, theoretically, under shear and compression strain in Cu_3_(1,3,5-tris(1*H*-pyrazol-4-yl)benzene)_2_^39^. Moreover, the presence of ligand vacancy also leads to the accessibility of hidden pore cavities^38^. Inspired by these findings, we explored the enhancement of the accessible surface area through the restoration of PBA crystals. Identical to most porous 3D MOFs, glass transformation reduces the pore accessibility in Cu[Fe]_2/3_-g, as demonstrated by N_2_ adsorption at 77 K (Fig. 3A). Negligible gas uptake with the Brunauer−Emmett−Teller (BET) surface area of 6.2 m^2^ g^−1^ was observed, a significant decrease from the Cu[Fe]_2/3_ (293 m^2^ g^−1^). Unlike other CPs/MOFs, where recrystallisation induced less than 100% recovery of the gas uptake compared with their pristine states would be expected^24^, we find an increase in the N_2_ uptake of Cu[Fe]_2/3_-c' with a BET surface area of 585 m^2^ g^−1^. The partial retention of the amorphous phase and the elimination of CN^−^ linkers during the mechanical milling process, described in the previous section, would generate local bypassing routes, resulting in a more accessible/connective porous network^33,38,39^.

### Semiconductivity of glass

The electron transport mechanism in PBA is composition-dependent. It can be classified as ranging from semiconductive in PBAs with a relatively narrow bandgap^40^ to nearest-neighbour hopping due to the localised 3*d* electron in Fe[Fe]_3/4_ and Berlin green with band insulator characteristics (10^−7^ to 10^−11^ S cm^−1^)^41^. A relatively low ligand-field splitting predicted for Cu-hexacyanoferrate among the metal hexacyanoferrates makes it a suitable candidate to elucidate the electronic conductivity in a glassy state^42^. The transport mechanism can be classified as class II mixed-valence compounds according to Robin−Day, where charges transfer between localised charge carriers through thermally activated electron hopping^40,43,44^. We evaluated the conductivity of Cu[Fe]_2/3_ in each state by the DC *I*–*V* method with a 2-contact probe under the Ar atmosphere to exclude the possible contribution from proton conductivity (Supplementary Figs. 23–25). All *I*–*V* curves show no sign of polarisation from migrated ions. In the experimental range, we found the dominance of semiconductive behaviour in Cu[Fe]_2/3_, Cu[Fe]_2/3_-g, and Cu[Fe]_2/3_-c' (Fig. 3B). With the presence of Fe^II/III^ mixed valency, Cu[Fe]_2/3_ exhibits the highest conductivity of 0.18 and 0.99 mS cm^−1^ at 30 and 50 °C, respectively. Slightly smaller conductivity values are observed in Cu[Fe]_2/3_-g and Cu[Fe]_2/3_-c' as Fe^III^ content diminished. Still, we find conductivity retention in Cu[Fe]_2/3_-g of 0.31 mS cm^−1^ at 50 °C, even in a glassy state. Comparing Cu[Fe]_2/3_-g and Cu[Fe]_2/3_-c', which share a comparable Fe^II^/Fe^III^ fraction, amorphous Cu[Fe]_2/3_-g shows a higher conductivity than crystalline Cu[Fe]_2/3_-c' throughout the experimental temperature regime. It is worth mentioning that the absence of long-range order usually suppresses the electronic conductivity in most semiconductive glasses due to the formation of trapped states in defect sites^44^. Cu[Fe]_2/3_-g, on the other hand, preserves short-range metal–ligand–metal connectivity while having a shorter pair distance between metal nodes (Cu–Fe of 4.94 Å) than its crystalline counterparts (5.03 Å), as suggested by PDF data (Fig. 2C). The close proximity of the redox hopping sites aids the continuity of the charge transport and plausibly compensates for the lack of long-range order^44,45^.

### Correlation between vacancies fraction and hydration on glass formation

Mechanical milling introduces shear and compression stress beyond the elastic stability limits of the crystals, resulting in a crystal-to-glass phase transition^46^. While the quantity of structural defects is difficult to control in other MOF/CP glasses, the tuneable vacancy/defect and compositional versatility in PBAs inspire us to generalise the correlation between vacancy fraction and hydration on glass-forming difficulty. Hereafter, PBAs with the nominal composition of M^II^[M'^III^(CN)_6_]·*n*H_2_O, M^II^[M'^III^(CN)_6_]_3/4_·*n*H_2_O, M^II^[M'^III^(CN)_6_]_2/3_·*n*H_2_O are denoted as M[M'], M[M']_3/4_, and M[M']_2/3_, respectively.

**Fig. 4:**
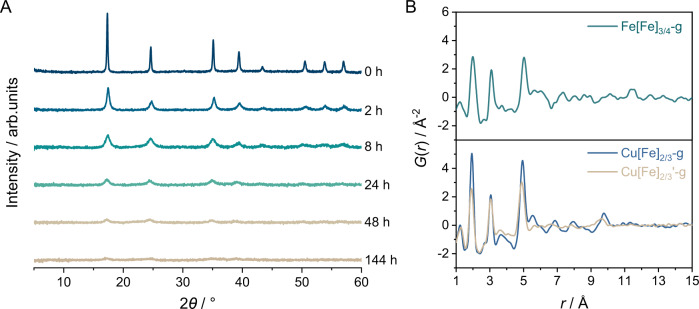
Effect of vacancies fraction and hydration. **A** PXRD of Fe[Fe]_3/4_ after mechanical milling for 0, 2, 8, 24, 48, and 144 h (λ = 1.5406 Å). **B** PDFs of Fe[Fe]_3/4_-g (144 h milling), Cu[Fe]_2/3_-g (2 h milling), and Cu[Fe]_2/3_'-g (2 h milling). Source data are provided as a Source Data file.

The presence of M'(CN)_6_ defect sites reduces connectivity and mechanical compression resistance, affecting the bulk modulus under pressure^15^. According to previous report^15^, the bulk moduli of Mn[Co]_2/3_, Cd[Co]_2/3_, and Mn[Pt] by second-order Birch-Murnaghan fitting^47,48^ are 6.5(7), 6.5(4), and 33(2) GPa, respectively. While the substitution of the metal between Mn[Co]_2/3_ and Cd[Co]_2/3_ negligibly contributed to the mechanical properties, bulk modulus of Mn[Pt] increased by a factor of 5 with the inclusion of additional M'(CN)_6_. Moreover, another work^49^ demonstrated that isostructural Mn[Pt] and Fe[Pt] also exhibit identical compressibility upon compression. Likewise, we expected that the intrinsic M'(CN)_6_ defects in PBA generally reduce the resistance to mechanical stress and should therefore shorten the milling time to achieve a fully non-crystalline state (Fig. 1). We extended the library toward the conventional Prussian blue with a quarter vacancy, Fe[Fe]_3/4_ (Supplementary Fig. 26, Supplementary Tables 5 and 6). Unlike Cu[Fe]_2/3_, which undergoes crystal–glass transformation under 2 h of ball milling, results show that Fe[Fe]_3/4_ remains highly crystalline under the same milling condition. The amorphous characteristics were then observed after 144 h of mechanical milling (Fig. 4A). We also found a glass transition temperature at *T*_*g*_ of 64.9 and 76.4 °C at a heating rate of 10 and 30 °C min^−1^, confirming the glassy nature of Fe[Fe]_3/4_-g (Supplementary Fig. 27). We acquired X-ray total scattering of the Fe[Fe]_3/4_-g at room temperature to evaluate the local structure. The PDF in Fig. 4B shows sharp features at a short scattering distance (*r* < 5.4 Å), confirming the preservation of Fe–C≡N–Fe connectivity in Fe[Fe]_3/4_-g with a nearby Fe–Fe pair distance of ca. 5.03 Å. In comparison, the Fe–Fe correlation in crystalline Fe[Fe]_3/4_ is 5.08 Å^4^. In higher *r*-space (Supplementary Fig. 28), detailed examination reveals quasiperiodic oscillations^20^ that continue to at least 35 Å even after 144 h of mechanical milling, much larger than that observed in Cu[Fe]_2/3_-g with 2 h of identical treatment (Supplementary Fig. 29). The presence of extended-range structural correlations mimics density fluctuation caused by topological and chemical ordering, which is also found in structurally disordered glassy AX_2_ inorganic systems, such as ZnCl_2_ and GeSe_2_^50,51^, and network-forming liquid/glassy MOFs/CPs^17,20^. Lack of sufficient void space and lower degree of freedom in Fe[Fe]_3/4_-g also affects the recrystallisation process. Only minor restoration of the Bragg diffraction is observed through the vapour-assisted recrystallisation at 80 °C (85 RH%) for 72 h (Fe[Fe]_3/4_-c, Supplementary Fig. 30).

We further evaluate the relationship between interstitial water and the degree of disorder in PBA. In contrast to melt-quenched glass, where no nuclei from the starting phase remain in the undercooled liquid, the glass produced by mechanical milling can exist in numerous thermodynamically unstable transient states^52^. Figure 4B and Supplementary Fig. 31 show PDF of glasses prepared from full hydrate (Cu[Fe]_2/3_-g) and dehydrate (Cu[Fe]_2/3_'-g) Cu[Fe]_2/3_. Weaker Cu[Fe]_2/3_'-g peaks amplitudes in short-range pairs distance (*r* < 5.3 Å) as well as peaks broadening at intermediate *r* region, as compared to Cu[Fe]_2/3_-g, is observed with a minor contraction of Cu–Fe pair distance (ca. 4.89 Å). Since the PDF provides the probability of finding an atom at a specific pair distance and displays it as a weighted histogram, these are particularly evident for a higher degree of disorder in Cu[Fe]_2/3_'-g^28,53^. The exclusion of interstitial water directly influences the framework integrity under mechanical stress under milling conditions. It is worth mentioning that the inclusion of solvent drastically changes the mechanical properties of porous materials^15,54^. Under compression, the bulk modulus of defective Mn[Co]_2/3_ doubles from 6.5(7) to 15.18(6) GPa with the inclusion of D_2_O^15^. Similar behaviour was also observed in Cu-based HKUST-1 and MOF-5, where the bulk modulus increased substantially in the penetrating pressure-transmitting medium compared to the nonpenetrating one^55,56^. Though our analysis has been intentionally simplified by neither acknowledging the inclusion of alkali cation nor the role of the Jahn-Teller effect^2^, the results show a direct correlation between the intrinsic defect fractions and the presence of interstitial water on the resistance upon glass-forming via mechanical stimuli.

### Elastic tensor analysis

**Fig. 5:**
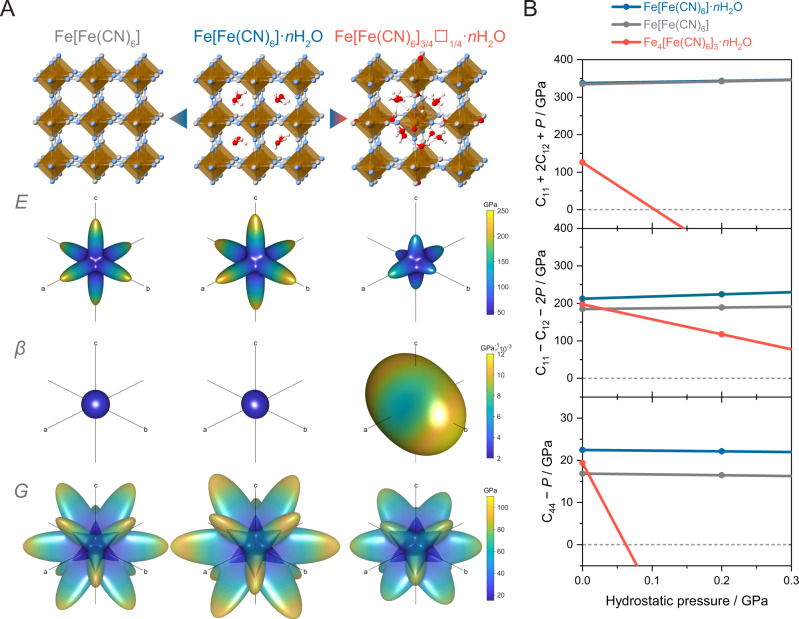
Elastic tensor analysis. **A** Spatial dependence of Young’s modulus (*E*), linear compressibility (*β*), and shear modulus (*G*) of Fe[Fe(CN)_6_], Fe[Fe(CN)_6_]·2H_2_O, and Fe[Fe(CN)_6_]_3/4_□_1/4_·3.5H_2_O. Since the *G* cannot be directly represented in 3D space, plots represent values range by enclosing the translucent maxima surface on an opaque minima surface. **B** Evolution of the stability criteria for compression (*λ* *=* C_11_ + 2C_12_ + *P*), (*λ* *=* C_11_ − C_12_ − 2 *P*), and shear (*λ* *=* C_44_ − *P*) of Fe[Fe(CN)_6_] (grey), Fe[Fe(CN)_6_]·2H_2_O (blue), and Fe[Fe(CN)_6_]_3/4_□_1/4_·3.5H_2_O (red) as a function of hydrostatic pressure. *C*_11_, *C*_12_, and *C*_44_ are the moduli for axial compression, dilation on compression, and shear, respectively. The dashed line indicates the stability limit for each mode (unstable when *λ* < 0). Fe[Fe(CN)_6_] represents the effect of the absence of interstitial water, while Fe[Fe(CN)_6_]_3/4_□_1/4_·3.5H_2_O describes the contribution of the hexacyanometallate vacancies. Source data are provided as a Source Data file.

To further understand the relationship between the composition of PBAs and the mechanical stability under applied stress, elastic components (*C*_ij_) were calculated based on energy-minimised structures of Fe[Fe(CN)_6_]·2H_2_O, Fe[Fe(CN)_6_], and Fe[Fe(CN)_6_]_3/4_·3.5H_2_O (Fig. 5A). These simplified series represent the defect-free PBAs, the influence of the absence of interstitial water, and the effect of quarter hexacyanometallate vacancy, respectively. Note that Fe[Fe(CN)_6_]_3/4_·3.5 H_2_O model adopts specifically ordered vacancies^2,4^. The defect-free Fe[Fe(CN)_6_]·2H_2_O demonstrates the highest ability to resist compression, linear stress, and shear deformation among the three, with an average Voigt-Reuss-Hill Bulk (*K*), Young’s (*E*), and shear (*G*) moduli of 114.7, 113.4, and 42.5 GPa, respectively (Supplementary Fig. 34)^57^. Elimination of interstitial water results in a minor to moderate decrease in stiffness of Fe[Fe(CN)_6_], where *K* = 111.6 GPa (−2.7%)*, E* = 98.7 GPa (−13.0%), and *G* = 36.5 GPa (−14.1%). Interstitial voids allow a certain degree of flexibility, thus lowering the framework stiffness. More details can be obtained by analysing the spatial dependence of mechanical properties calculated from the elastic component (Fig. 5A, Supplementary Figs. 36–43, and Supplementary Table 7)^58,59^. The theoretical Young’s modulus (*E*) of Fe[Fe(CN)_6_]·2H_2_O shows an *E*_max_ of 274.4 GPa along the primary axis where the acting stress is parallel to the metal–ligand connectivity. The *E*_min_ of 51.4 GPa can be found at diagonal positions, resulting in an anisotropy value (*E*_max_/*E*_min_) of 4.8. *E*_max_ and *E*_min_ were reduced to 217.3 and 48.2 GPa in Fe[Fe(CN)_6_] with a smaller degree of anisotropy of 4.5 since the alignment of interstitial water lightly disturbed the symmetry of Fe[Fe(CN)_6_]·2H_2_O. The exclusion of 25% occupancy of hexacyanometallate in Fe[Fe(CN)_6_]_3/4_·3.5H_2_O results in a dramatic decrease in the average *K* and *E* values to 37.3 (−67.5%) and 84.4 (−25.6%) GPa, respectively (Fig. 5A, Supplementary Figs. 34, and  44–47). The presence of vacancy sites reduces the framework connectivity, which significantly lowers the resistance toward applied stress. Identical behaviour was also observed in other MOFs/CPs, where linkers or metal vacancies result in higher compressibility and lower mechanical stability under applied stress^60,61^. A higher degree of anisotropy was also observed in spatial dependence of *E*, *β* (linear compressibility), and *G* (shear modulus). Despite a low *G*_max_ value of 78.6 GPa, Fe[Fe(CN)_6_]_3/4_·3.5 H_2_O exhibits a relatively high *G*_min_ (18.8 GPa) and low *G* anisotropy of 4.2, which results in a moderate average *G* of 37.6 (−11.5%). The hydrogen-bonded network constructed from coordinated waters binds to the coordinatively unsaturated metal sites, and interstitial water contributes to resisting the shear deformation^15^. Although the calculated moduli are larger than the experimental values found for PBAs^15,62^, the trends highlight the opportunity to understand the mechanical crystal–glass transformation process through control of the PBA composition.

The fundamental cause of the crystal-to-glass transition under mechanical stress was further elucidated by monitoring the evolution of elastic constants of PBAs under hydrostatic compression (Fig. 5B)^46,54^. Under applied compression, the stability criteria of Fe[Fe(CN)_6_]_3/4_□_1/4_·3.5 H_2_O diminishes drastically in all modes due to the framework softening. However, the origin of elastic instability of the framework appears to be initiated by the shear-mode deformation (*C*_44_ < 0), which is estimated to begin at *P* = 0.06 GPa. In contrast, the result indicates that the deformation of Fe[Fe(CN)_6_]·2H_2_O and Fe[Fe(CN)_6_] can only be triggered by the shear mode softening at a pressure much higher than that of Fe[Fe(CN)_6_]_3/4_□_1/4_·3.5 H_2_O. These results highlight the impact of hexacyanometallate vacancy on the mechanical-induced crystal–glass transformation process. Moreover, compared between Fe[Fe(CN)_6_]·2 H_2_O and Fe[Fe(CN)_6_], the presence of interstitial water delays the phase transition onset pressure by altering the shear modulus of the unstressed material.

### Mechanical properties of glass

**Fig. 6:**
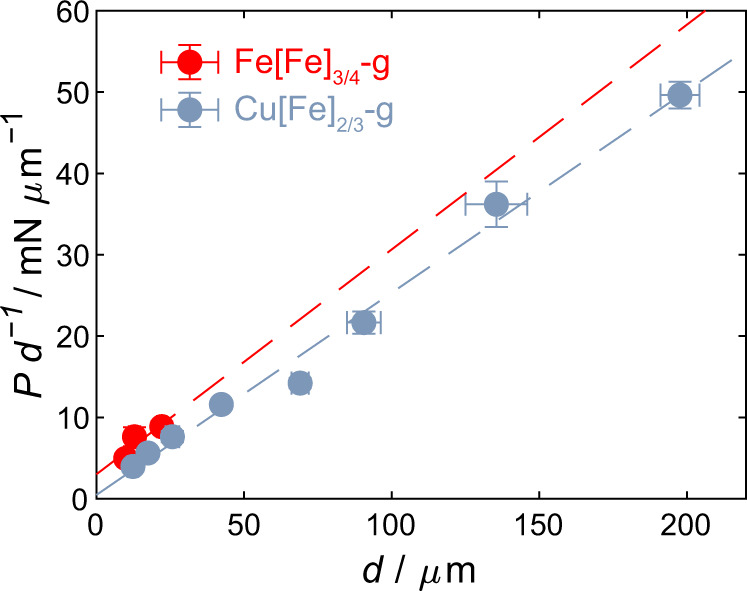
Mechanical properties of glass. Bernhardt plot of the Vickers indentation data for the Fe[Fe]3/4-g (red) and Cu[Fe]2/3-g (blue) monolith samples. The linear fit is used to extract the constants in Eq. 3 (see Methods) and, from the slope, ultimately to provide the Vickers hardness at infinite load as presented in Supplementary Table 8. Error bars are expressed as the arithmetic mean ± standard deviations. Source data are provided as a Source Data file.

We then tested the bulk mechanical properties of Fe[Fe]_3/4_-g and Cu[Fe]_2/3_-g glass monoliths (Supplementary Table 8). Through indentation measurements, the Vickers hardness at infinite load (see Methods for details) was found to be 0.51 GPa and 0.46 GPa for the Fe[Fe]_3/4_-g and Cu[Fe]_2/3_-g samples, respectively (Fig. 6). Furthermore, based on the load-depth curves, we estimated the reduced elastic moduli for the monoliths to be 7.6 GPa and 5.7 GPa, respectively. Using Archimedes’ principle and the ultrasonic echography method, we determined the densities and longitudinal sound velocity for both monoliths and the transversal sound velocity for the Cu[Fe]_2/3_-g monolith. This provided densities of around 2 g cm^−3^ and longitudinal velocities just below 3000 m s^−1^. This ultimately yielded longitudinal moduli in the range of 16 to 17 GPa, with a slightly higher value for the Fe[Fe]_3/4_-g monolith. Based on the performed measurements, it was possible to calculate all mechanical moduli for the Cu[Fe]_2/3_-g monolith (assuming isotropicity), yielding Young’s, shear, and bulk moduli of *E* = 12.6 GPa, *G* = 4.9 GPa, and *B* = 9.7 GPa, respectively, while the Poisson’s ratio was found to be *ν* = 0.28 (Supplementary Table 8). Generally, we note that bulk mechanical properties are similar for the two glass monoliths, suggesting largely similar bonding types and energies in the two systems. Comparing these results to previous experimental measurements on MOF glasses, namely zeolitic imidazolate framework (ZIF) glasses, both similarities and differences are apparent. While the hardness is relatively similar for the two families (ca. 0.5 GPa)^63^, it is notable that both density (ca. 1.4 to 1.6 g cm^−3^ for ZIF glasses)^64,65^ and mechanical moduli (*E* ca. 4 to 8 GPa for ZIF glasses)^66,67^ appear to be higher for the Cu[Fe]_2/3_-g PBA when considering the echography data, but more similar when considering the reduced elastic modulus data. Finally, comparing the Poisson’s ratio of glassy PBAs and ZIFs, we find that the Cu[Fe]_2/3_-g has a significantly smaller *ν* of ca. 0.28 in contrast to glassy ZIF-62, which features *ν* in the range of 0.35 to 0.45^63,68^.

In summary, the foregoing results demonstrate the first confirmation of glassy states in Prussian blue analogues. Mechanical induced crystal–glass transformation successfully converts crystalline PBAs to amorphous solids, exhibiting glass transition behaviour. Synchrotron X-ray total scattering results with following PDF analysis confirm the preservation of short-range order with the retention of metal–ligand–metal connectivity. Apart from the physical structure, partial alteration of the oxidation state from Fe^III^ to Fe^II^ was recognised with CN^−^ elimination to satisfy electroneutrality. Furthermore, the observation of an enhancement in N_2_ gas uptake in recrystallised Cu[Fe]_2/3_-c (585 m^2^ g^−1^) and the demonstration of electronic conductivity in glassy Cu[Fe]_2/3_-g (0.31 mS cm^−1^ at 50 °C) also initiated a foundation toward the functionality of PBA glasses. Finally, we correlate the contribution of intrinsic vacancy and interstitial water to mechanical properties. Intrinsic stoichiometry plays a dominant role in structural rigidity and thus increases the requirement for mechanical stress to initiate the crystal–glass transformation. The presence of interstitial water also contributes to the stiffness of the framework. This work thus introduces PBA as a new family of CP/MOF glasses and establishes the foundation for the fundamental understanding of mechanical-induced glass.

## Methods

### Synthesis of Prussian blue analogues

The K_2*x*/3_Cu^II^[Fe^II^_*x*_Fe^III^_*1−x*_(CN)_6_]_2/3_□_1/3_·*x*H_2_O (Cu[Fe]_2/3_) was synthesised via reported precipitation method^5,6^. 120 ml of 0.2 M CuSO_4_ was introduced dropwise to a vigorously stirring 120 ml of 0.1 M K_3_Fe(CN)_6_. After 6 h of reaction, the precipitate was washed and centrifuged multiple times and dried in an oven at 60 °C for 12 h. The exact composition of Cu[Fe]_2/3_ is K_0.32_Cu[Fe(CN)_6_]_0.63_·*n*H_2_O, where the Fe:Cu and K:Fe ratios were obtained from Energy Dispersive X-ray Fluorescence (EDXRF, Supplementary Table 1) and the Fe^II^ content from ^57^Fe Mössbauer spectroscopy (Supplementary Fig. 19 and Supplementary Table 2), respectively. Fe^III^[Fe^II^(CN)_6_]_3/4_□_1/4_·*x*H_2_O (Fe[Fe]_3/4_) was purchased from Alfa Aesar and used without further purification.

### Preparation of mechanically induced glasses

Solvent-free mechanical milling was conducted by introducing 500 mg of crystalline TBA to a planetary ball-milling apparatus (Fritsch, Pulverisette 7) with a zirconia vessel (20 ml) and balls (10 balls with a diameter of 10 mm) in an Ar-filled glove box. The rotation speed of the solar disc was set to 400 rpm with an alternate milling period of 5 min and a pause of 5 min to avoid overheating. The alternate period of 10 min was utilised for milling time over 4 h. Milled samples were collected and kept in an Ar-filled glovebox. For Fe[Fe]_3/4_-g, ball milling was conducted for 144 h. In situ temperature-probing during the mechanical milling process was conducted using Fritsch Easy GTM-bowls with Fritsch Pulverisette 7.

### Vapour-assisted annealing

Samples were placed at 80 °C, 85 RH% for 72 h. The relative humidity and temperature were controlled by an Espec Corp. SH-221 incubator.

### Material characterisations

Powder X-ray diffraction (PXRD) patterns were collected using a Rigaku MiniFlex with a CuKα anode. Thermogravimetric analysis (TGA) results were measured with a heating rate of 10 °C min^−1^ under flowing Ar using a Rigaku Thermo plus TG 8121. Scanning electron microscope (SEM) images were taken using a Hitachi SU5000 instrument after Osmium plasma-chemical vapour deposition for 5 s. Cross-section samples were prepared under liquid nitrogen. Differential Scanning Calorimetry (DSC) was collected using the Netzsch Sirius 3500 in an N_2_ atmosphere (Al crucible). IR spectra were obtained with a Thermo Scientific Nicolet Summit FT-IR equipped with a diamond ATR accessory. Results of Energy Dispersive X-ray Fluorescence (EDXRF) experiments were collected using the Rigaku NEX CG/LC2 instrument in a He atmosphere.

### X-ray total scattering

Each sample was filled in a Lindemann glass capillary with a diameter of 2 mm and sealed inside an Ar-filled glove box. The synchrotron X-ray total scattering was collected on the BL04B2 beamline at the Super Photon ring-8 GeV (SPring-8, Hyogo, Japan) with four CdTe and two Ge detectors covering the Q range up to 25 Å^−1^ (61.377 keV; λ = 0.2020 Å). The collected scattering data was applied absorption, background, and Compton scattering corrections, then normalised to give the Faber−Ziman total structure factor S(Q). The pair distribution functions were calculated by Fourier transforming the S(Q) with a Lorch modification function^26,69,70^. Rietveld refinement of synchrotron X-ray Diffraction was analysed using Rigaku PDXL2 software.

### Extended X-ray absorption fine-structure spectroscopy

Each sample was mixed with boron nitride and pressed into pellets with a diameter of 10 mm (0.5 mm thick) and sealed inside an Ar-filled glove box. The synchrotron X-ray absorption spectra in the energy region of the Fe K-edge were collected in transmission mode on the BL01B1 beamline at the Super Photon ring-8 GeV (SPring-8, Hyogo, Japan). Fourier transformation was *k*3-weighted in the *k* range of 3.0 to 14 Å^−1^. The data processing and fitting were performed with Athena and Arthemis software.

### Mössbauer spectroscopy

Zero-field^57^ Fe Mössbauer spectra were measured at room temperature and performed with a Wissel MVT-1000 Mössbauer spectrometer, with a^57^ Co/Rh source in transmission mode. All Mössbauer spectra were calibrated at room temperature with α-Fe and were fitted using the MossA software package^71^.

### Electronic conductivity

Conductivity measurements were performed using linear sweep voltammetry techniques using an EC-Lab VSP-300 (Bio-Logic Science Instruments SAS). Powder samples (ca. 50 mg) were pressed at 600 kgf for 1 min using a 5 mm die. The pellet was sandwiched between two gold electrodes. I–V curves were collected between −2 to 2 V at 100 mV s^−1^.

Electronic conductivity was calculated using the following equation:

1 $${{{{{\rm{\sigma }}}}}}=\frac{L}{\left(\frac{V}{I}\right)\times \pi {r}^{2}}$$

*L* represents the thickness of the pellet, *V* is the potential, *I* is the current, and *r* is the pellet radius.

### N_2_ gas adsorption

Gas adsorption isotherms were collected by a BELSORP-mini with a cryostat system. The Brunauer−Emmett−Teller (BET) surface area was calculated using the N_2_ adsorption isotherm at 77 K. Each sample was activated at 70 °C under vacuum for 24 h.

### Computational method

The elastic modulus calculations were performed using the Vienna Ab Initio Simulation Package (VASP)^72–75^. The Perdew-Burke-Ernzerhof (PBE) functional^76–78^ was employed for electronic structure calculation, with the wavefunctions being constructed using the projector-augmented wave method^79,80^. For the correction of dispersion interaction, we used the well-developed D3 method developed by Grimme et al.^81^. The electronic convergence criterion of 10^−6^ eV was set for self-consistency, while the geometry convergence criterion was set at 1.0 × 10^−2^ eV/Å (for small structures) or 2.0 × 10^−2^ eV/Å (for large structures). Subsequently, the Hessian matrix was analysed using a numerical approximation method with two distortions done on each degree of freedom (including the unit cell). Spatial moduli were illustrated by either AnisoVis (https://github.com/DaveHealy-Aberdeen/AnisoVis)^59^ or the elastic tensor analysis ELATE (http://progs.coudert.name/elate)^58^. The elastic instability was evaluated through the Born stability criteria^46,54^.

### Elastic stability

Since the PBAs are cubic, the elastic stability criteria under hydrostatic pressure *P* are (i) *C*_11_ + 2*C*_12_ + *P* ≥ 0, (ii) *C*_11_ − 2*C*_12_ ≥ 2 *P*, and (iii) *C*_44_ ≥ 0^46,54^. Conditions (i) and (ii) correspond to the compression mode, while (iii) represents shear mode deformation. *C*_11_, *C*_12_, and *C*_44_ are the moduli for axial compression, dilation on compression, and shear, respectively.

### Mechanical characterization

The glass monolith samples Fe[Fe]_3/4_-g and Cu[Fe]_2/3_-g were first polished using SiC grinding papers and, subsequently, using 250 nm diamond paste, both employing *n*-hexane as the lubrication medium. The Vickers hardness was then determined using a Struers Duramin 40 instrument equipped with a Vickers diamond tip. Indentation experiments were performed using loads (*P*) of 49.0, 98.0, and 196.1 mN (5, 10, and 20 gf, respectively) for the Fe[Fe]_3/4_-g monolith and additional loads of 490.3, 980.7, 1961.3, 4903.3, and 9806.65 mN (50, 100, 200, 500, and 1000 gf, respectively) for the Cu[Fe]_2/3_-g monolith. Five indentations were performed for each load, and a creep time of 15 s was used for all indentation experiments. The diagonal lengths (*d*) of the produced indents were then recorded based on optical microscopy images, and the Vickers hardness (*H*_V_) was calculated as,

2 $${H}_{{{{{{\rm{V}}}}}}}=1.854\frac{P}{{d}^{2}}$$

Based on the relation suggested by Bernhardt^82^, we fitted the obtained hardness vs. load data using the relation,

3 $$\frac{P}{d}={a}_{1}+{a}_{2}d,$$

where *a*_1_ and *a*_2_ are empirical constants describing the extent of the indentation size effect and the hardness at infinite load, respectively. By small rearrangement, the Vickers hardness at infinite load may be obtained as,

4 $${H}_{V,{{\infty }}}=\mathop{{{{{{\rm{lim}}}}}}}\limits_{d\to \,{{\infty }}}1.854\frac{P}{{d}^{2}}=1.854{a}_{2}$$

Additional indentation experiments were performed using a Nanovea CB500 indenter, equipped with a diamond Vickers tip and a depth sensor, to obtain the stiffness of the two glass samples. Five indents at a load of 196.1 mN (20 gf) were performed. The stiffness (*S*) was obtained from the unloading curve of the load/depth response by fitting a linear curve to the unloading response between 80 and 95% of the maximum load. The diagonal lengths (*d*) of the produced indents were then obtained, and finally, the reduced elastic modulus (*E*_r_) was calculated as^83^,

5 $${E}_{r}=\beta \frac{S}{d}\sqrt{\frac{\pi }{2}}$$

where *β* = 1.0124 is a tip-specific constant for the Vickers geometry.

Sound velocities of the glass monoliths were measured using an Olympus 38DL Plus ultrasonic thickness gauge. The longitudinal velocity (*V*_L_) was measured for both the Fe[Fe]_3/4_-g and Cu[Fe]_2/3_-g samples, while the transversal velocity (*V*_T_) was only measured for the latter due to a weak sample signal for the former. The density (*ρ*) of the monoliths was determined by the Archimedes’ principle by measuring the mass of monoliths submerged in *n*-hexane at room temperature as,

6 $$\rho =\frac{{\rho }_{{{{{{\rm{hexane}}}}}}}{m}_{{air}}}{{m}_{{air}}-{m}_{{sub}}}$$

where *ρ*_hexane_ = 0.6606 g cm^−3^, *m*_air_ is the mass of the sample measured in air, and *m*_sub_ is the mass of the sample while submerged. Finally, combining the measured densities and sound velocities provided the longitudinal modulus (*L*) for both the Fe[Fe]_3/4_-g and Cu[Fe]_2/3_-g monoliths, as well as Young’s (*E*), shear (*G*), and bulk (*B*) moduli and the Poisson’s ratio (*ν*) for the Cu[Fe]_2/3_-g monolith.

7 $$L={V}_{{{{{{\rm{L}}}}}}}^{2}\rho$$

8 $$E=2G(1+\upsilon )$$

9 $$G={V}_{{{{{{\rm{T}}}}}}}^{2}\rho$$

10 $$B=\frac{E}{3(1-2\upsilon )}$$

11 $$\upsilon =\frac{{V}_{{{{{{\rm{L}}}}}}}^{2}-2{V}_{T}^{2}}{2({V}_{{{{{{\rm{L}}}}}}}^{2}-{V}_{T}^{2})}$$

## Supplementary information

Supplementary information

## Data Availability

The data generated in this study are provided in the Supplementary Information and Source Data file. Additional data are available from the corresponding author on request. Source data are provided with this paper.

## Source data

Source Data

## Code availability

The code used for the elastic tensor analysis within this paper is available from the corresponding author on request.
