# Supplementary material for: Exploration of glassy state in Prussian blue analogues
Source: Nat Commun. 2022 Jul 12;13:4023. doi: 10.1038/s41467-022-31658-w (PMC9276687; doi:10.1038/s41467-022-31658-w)
Supplement: Supplementary file 1 — Supplementary information [file 41467_2022_31658_MOESM1_ESM.pdf]

Supplementary Information

Exploration of glassy state in Prussian blue analogues

N. Ma *et al.*

*Corresponding author. Email: horike@icems.kyoto-u.ac.jp (Satoshi Horike)

Supplementary Tables

Supplementary Table 1. Fe and Cu mass percent of $Cu[Fe]_{2/3}$ from Energy Dispersive X-ray Fluorescence (EDXRF).

	Mass (%)	Standard deviation	Fe:Cu mol ratio
Cu	63.9	0.1	0.63
Fe	35.1	0.1	

Supplementary Table 2. Room temperature ^{57}Fe Mössbauer spectral fitting parameters.

	Component	IS (mm/s)	QS (mm/s)	Fraction (%)
$Cu[Fe]_{2/3}$	Fe^{II}	–0.088	0.179	51.1
	Fe^{III}	–0.159	0.556	48.9
$Cu[Fe]_{2/3}$-g	Fe^{II}	–0.088	0.168	67.0
	Fe^{III}	0.258	0.790	33.0
$Cu[Fe]_{2/3}$-c	Fe^{II}	–0.086	0.143	73.5
	Fe^{III}	0.318	0.727	26.5
$Cu[Fe]_{2/3}$-c'	Fe^{II}	–0.083	0.172	66.9
	Fe^{III}	0.309	0.745	33.1

Supplementary Table 3. C and N weight percentage of $Cu[Fe]_{2/3}$, $Cu[Fe]_{2/3}$-g, and $Cu[Fe]_{2/3}$-c excluding water content. The values are calculated from elemental analysis.

	Element	Weight (%)	Standard deviation
$Cu[Fe]_{2/3}$	C	23.7	0.34
	N	28.4	0.77
$Cu[Fe]_{2/3}$-g	C	22.7	0.20
	N	27.0	0.56
$Cu[Fe]_{2/3}$-c	C	22.2	0.51
	N	26.6	0.34

Supplementary Table 4. Fe K-edge EXAFS data fitting results.

	Path	nleg	Degeneracy	R (Å)	σ^2 (Å^2)	E_0 (eV)
$Cu[Fe]_{2/3}$	Fe–C	2	6.0	1.93	0.004	−2.691
	Fe–N	2	6.0	3.06	0.031	−2.691
	Fe–N	3	12.0	3.06	0.007	−2.691
	Fe–N	4	6.0	3.06	0.005	−2.691
$Cu[Fe]_{2/3}$-g	Fe–C	2	4.4	1.92	0.005	−2.797
	Fe–N	2	4.4	3.01	0.027	−2.797
	Fe–N	3	8.8	3.01	0.007	−2.797
	Fe–N	4	4.4	3.01	0.003	−2.797
$Cu[Fe]_{2/3}$-c	Fe–C	2	5.4	1.91	0.005	−4.093
	Fe–N	2	5.4	3.03	0.046	−4.093
	Fe–N	3	10.8	3.03	0.012	−4.093
	Fe–N	4	5.4	3.03	0.004	−4.093

Supplementary Table 5. Room temperature ^{57}Fe Mössbauer spectral fitting parameters of Fe[Fe]$_{3/4}$.

	Component	IS (mm/s)	QS (mm/s)	Fraction (%)*
Fe[Fe]$_{3/4}$	Fe^{II}	–0.143	0.118	48.1
	$Fe^{III,\,a}$	0.410	0.001	17.7
	$Fe^{III,\,b}$	0.410	0.270	22.6
	$Fe^{III,\,c}$	0.410	0.627	11.6

*Fe^{III}/Fe^{II} ratio of $Fe^{III}[Fe^{II}(CN)_6]_{3/4}\square_{1/4}\cdot nH_2O$ measured from ^{57}Fe Mössbauer spectroscopy is found to range from 0.99 to 1.13 (1.08 for this work) instead of the theoretical value of 1.33 due to the deviation of the *f*-factor between Fe^{II}–C bonding and the average of the Fe^{III}–N, Fe^{III}–O, and Fe^{III}–vacancy bonding.[1]

Supplementary Table 6. C and N weight percentage of Fe[Fe]$_{3/4}$ excluding water content. The values are calculated from elemental analysis.

	Element	Weight (%)	Standard deviation
Fe[Fe]$_{3/4}$	C	27.6	0.58
	N	35.5	0.55

Supplementary Table 7. Variations of elasticity for $Fe[Fe(CN)_6]\cdot nH_2O$, $Fe[Fe(CN)_6]$, and $Fe_4[Fe(CN)_6]_3\cdot nH_2O$.

		$Fe[Fe(CN)_6]\cdot nH_2O$	$Fe[Fe(CN)_6]$	$Fe_4[Fe(CN)_6]_3\cdot nH_2O$
Young's modulus	E_{max} / GPa	247.4	217.3	169.0
	E_{min} / GPa	551.4	48.2	48.9
	A	4.8	4.5	3.5
Linear compressibility	β_{max} / TPa^{-1}	3.0	3.0	11.8
	β_{min} / TPa^{-1}	2.7	3.0	6.6
	A	1.1	1.0	1.8
Shear modulus	G_{max} / GPa	103.7	92.4	78.6
	G_{min} / GPa	16.7	16.9	18.8
	A	6.2	5.5	4.2
Poisson's ratio	v_{max}	0.78	0.77	0.58
	v_{min}	0.03	0.05	−0.25
	A	29.3	16.0	∞

Supplementary Table 8. Density (ρ), longitudinal velocity (V_L), Vickers hardness at infinite load ($H_{V,\infty}$), reduced elastic modulus (E_r) from indentation experiments, and longitudinal modulus (L) of Fe[Fe]$_{3/4}$-g and Cu[Fe]$_{2/3}$-g monolith samples as well as transversal velocity (V_T), Young's modulus (E), shear modulus (G), bulk modulus (B), and Poisson's ratio (ν) for the Cu[Fe]$_{2/3}$-g monolith. n.d. indicates that property could not be determined.

	Fe[Fe]$_{3/4}$-g	Cu[Fe]$_{2/3}$-g
ρ (g cm^{-3})	2.04	1.95
V_L (m s^{-1})	2950	2891
V_T (m s^{-1})	n.d.	1586
$H_{V,\infty}$ (GPa)	0.51	0.46
E_r (GPa)	7.6	5.7
L (GPa)	17.8	16.3
E (GPa)	n.d.	12.6
G (GPa)	n.d.	4.9
B (GPa)	n.d.	9.7
ν (-)	n.d.	0.28

Supplementary Figures

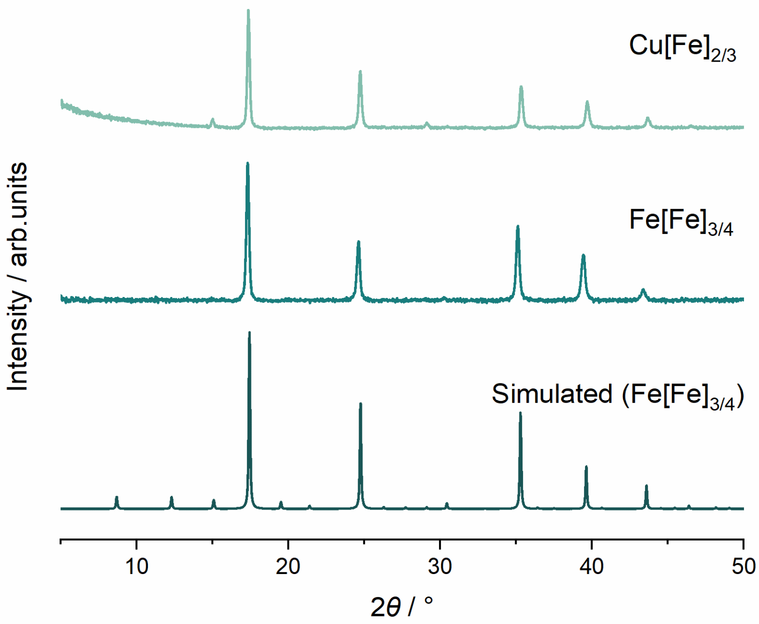

Supplementary Figure 1. PXRD patterns of $Cu[Fe]_{2/3}$, $Fe[Fe]_{3/4}$, and simulated $Fe[Fe]_{3/4}$ (λ = 1.5406 Å). Source data are provided as a Source Data file.

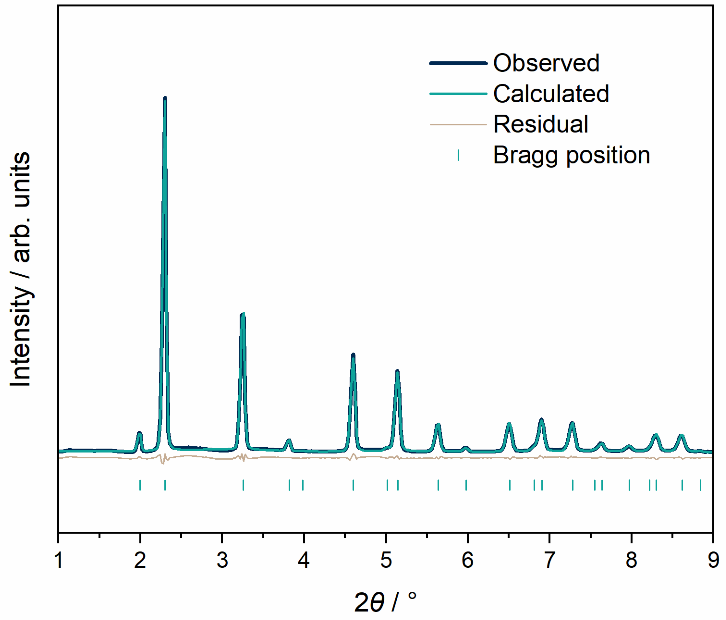

Supplementary Figure 2. Rietveld refinement of $Cu[Fe]_{2/3}$ (λ = 0.2020 Å). Source data are provided as a Source Data file.

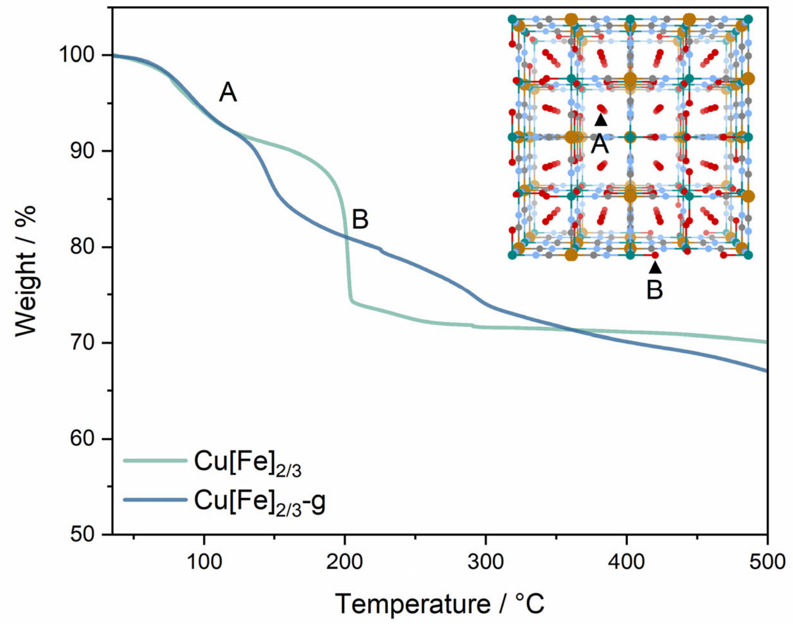

Supplementary Figure 3. TGA profiles of Cu[Fe]$_{2/3}$ and Cu[Fe]$_{2/3}$-g under Ar (10 °C min^{-1}). Inset scheme displays (A) zeolitic water and (B) coordinate water. Source data are provided as a Source Data file.

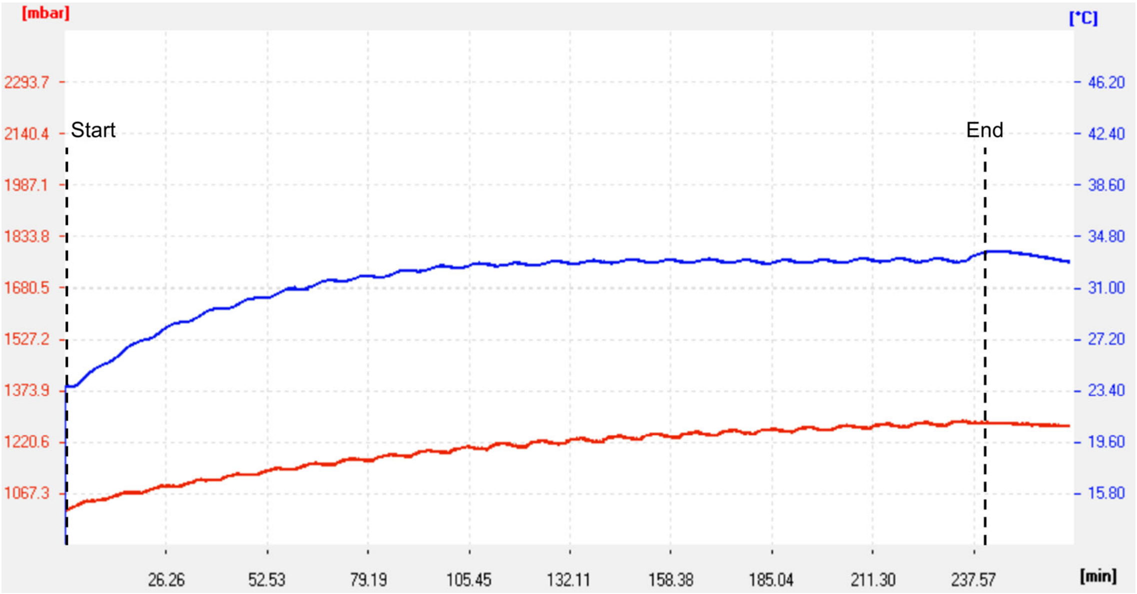

Supplementary Figure 4. In situ temperature-probing during the mechanical milling process of Cu[Fe]$_{2/3}$.

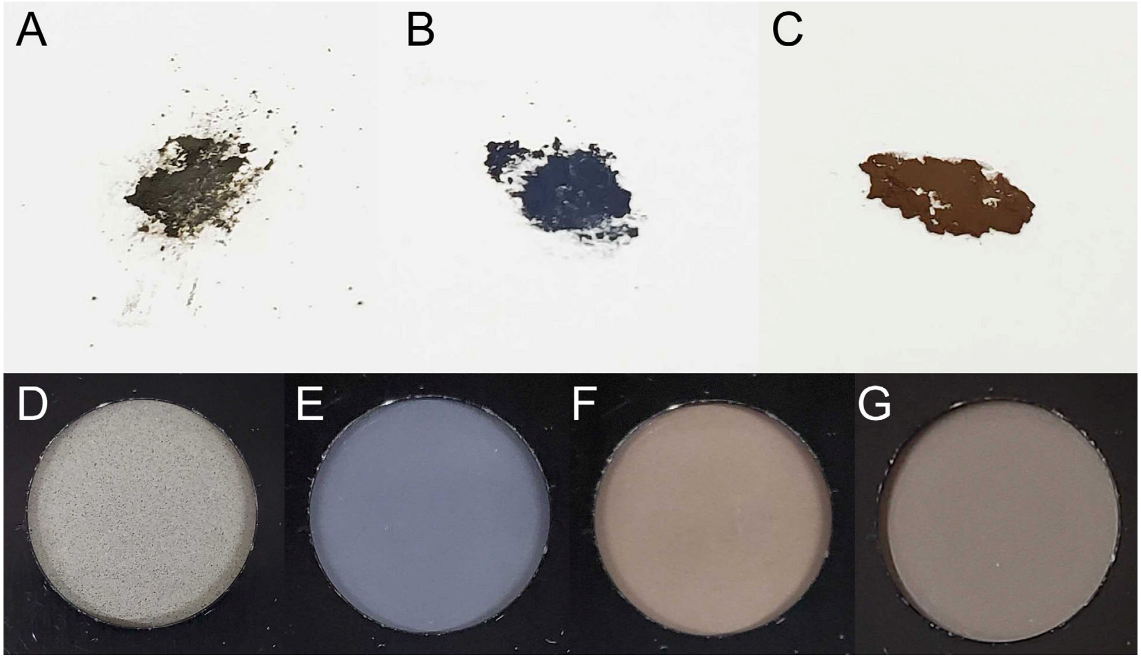

Supplementary Figure 5. Optical images of (A) Cu[Fe]$_{2/3}$, (B) Cu[Fe]$_{2/3}$-g, and (C) Cu[Fe]$_{2/3}$-c as well as samples mixed with 95 wt% CaF_2 to emphasise the colour change of (D) Cu[Fe]$_{2/3}$, (E) Cu[Fe]$_{2/3}$-g, (F) Cu[Fe]$_{2/3}$-c, and (G) Cu[Fe]$_{2/3}$-c'.

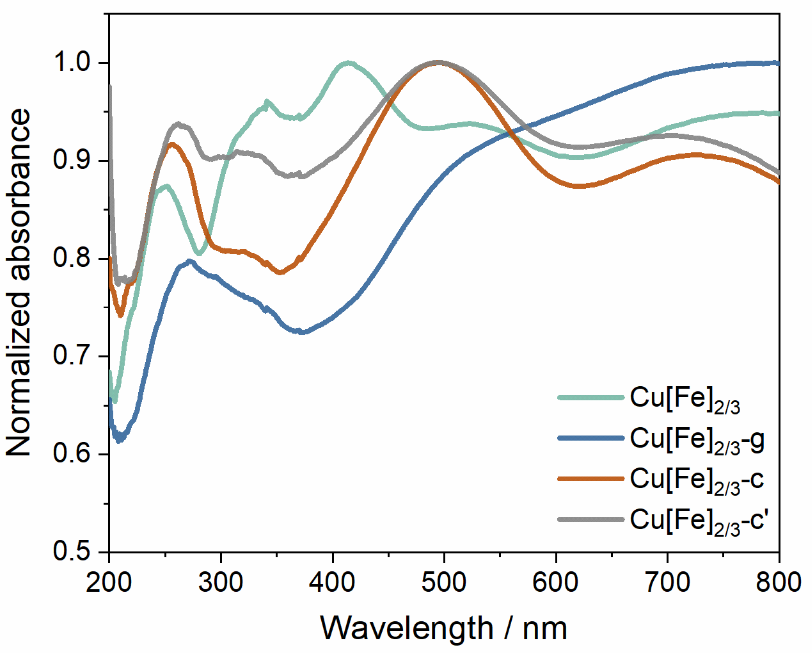

Supplementary Figure 6. Diffuse-reflectance UV-Vis spectra of Cu[Fe]$_{2/3}$, Cu[Fe]$_{2/3}$-g, Cu[Fe]$_{2/3}$-c, and Cu[Fe]$_{2/3}$-c'. Source data are provided as a Source Data file.

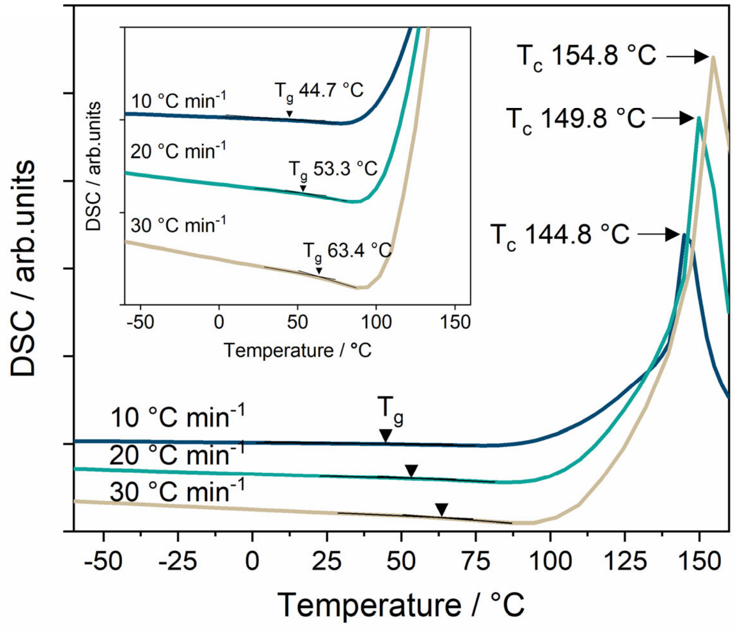

Supplementary Figure 7. Close system DSC profiles under Ar atmosphere of Cu[Fe]$_{2/3}$-g at the heating rate of 10, 20, and 30 °C min^{-1}. Source data are provided as a Source Data file.

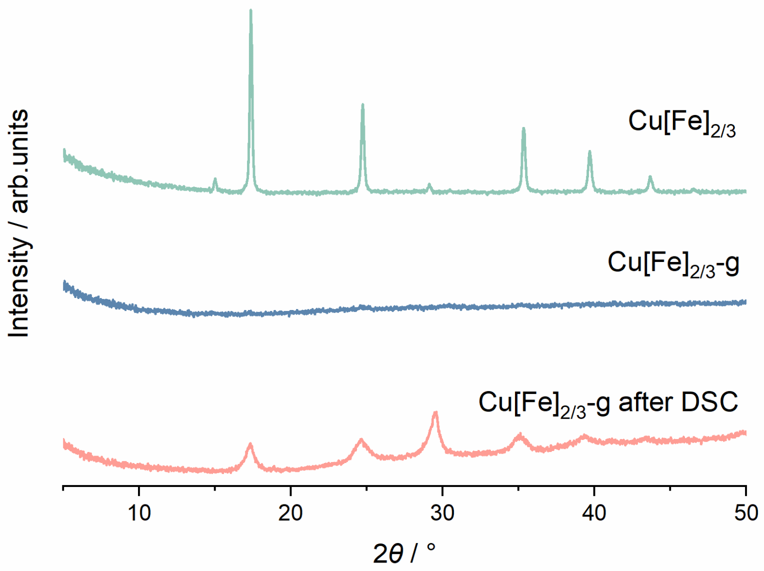

Supplementary Figure 8. PXRD patterns of Cu[Fe]$_{2/3}$, Cu[Fe]$_{2/3}$-g, and Cu[Fe]$_{2/3}$-g after DSC measurement (λ = 1.5406 Å). Source data are provided as a Source Data file.

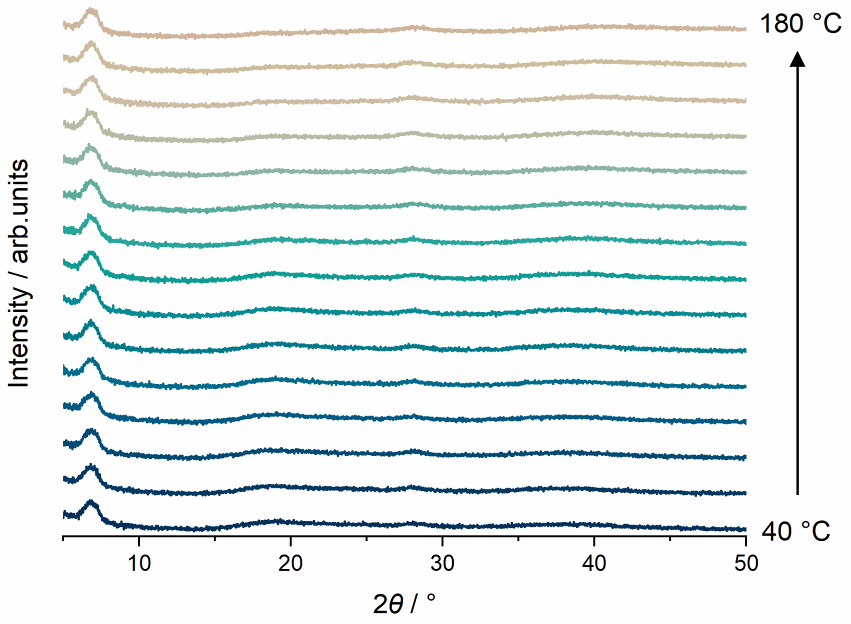

Supplementary Figure 9. Ar-flow variable temperature PXRD patterns of Cu[Fe]$_{2/3}$-g from 40 to 180 °C (λ = 1.5406 Å). The temperature was held for 30 min before each measurement. The peak at the 2θ of 6.9 ° is corresponding to the cell background. Source data are provided as a Source Data file.

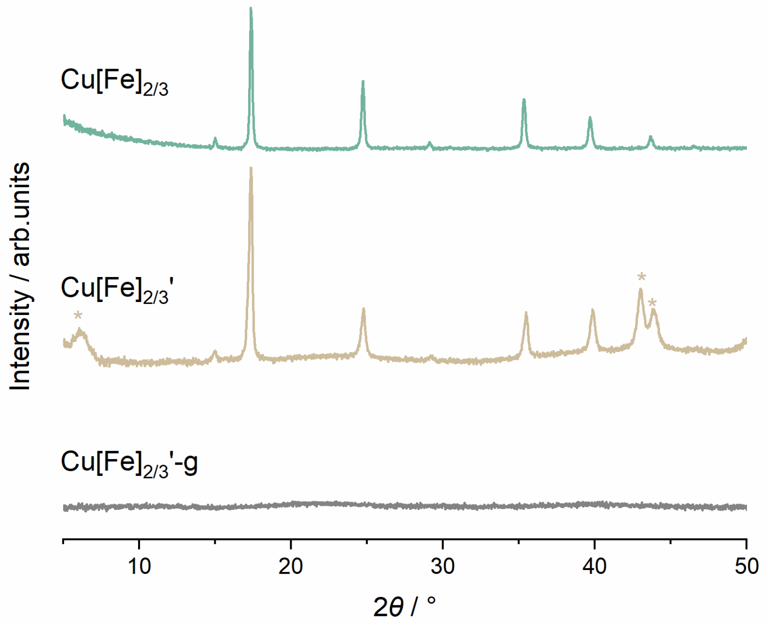

Supplementary Figure 10. PXRD patterns of Cu[Fe]$_{2/3}$, Cu[Fe]$_{2/3}$', and Cu[Fe]$_{2/3}$'-g. Peaks with (*) is corresponding to the Ar cell background (λ = 1.5406 Å). Source data are provided as a Source Data file.

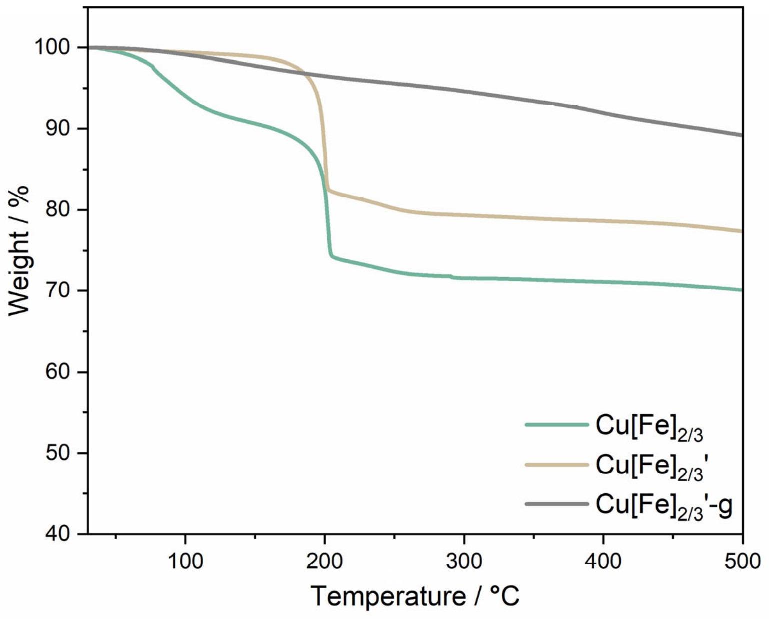

Supplementary Figure 11. TGA profiles of $Cu[Fe]_{2/3}$, $Cu[Fe]_{2/3}'$, and $Cu[Fe]_{2/3}'$-g. Source data are provided as a Source Data file.

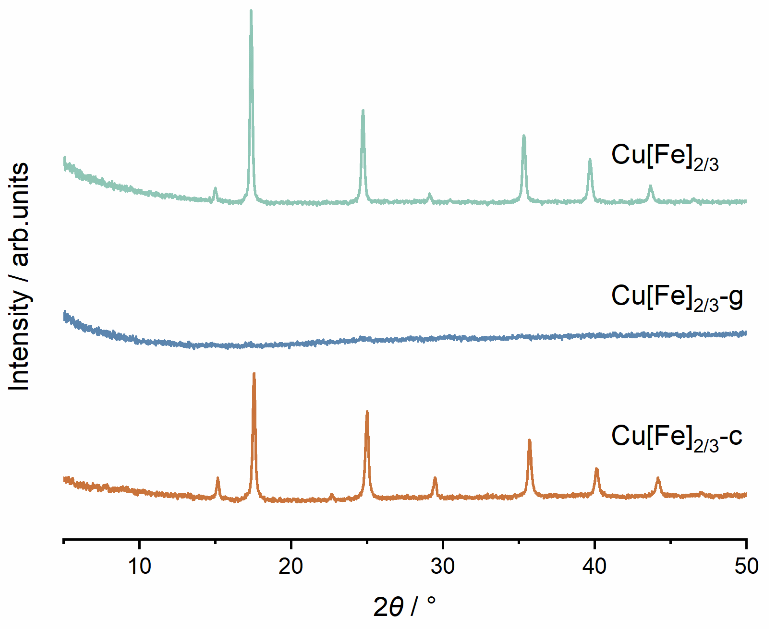

Supplementary Figure 12. PXRD patterns of $Cu[Fe]_{2/3}$, $Cu[Fe]_{2/3}$-g, and $Cu[Fe]_{2/3}$-c (λ = 1.5406 Å). Source data are provided as a Source Data file.

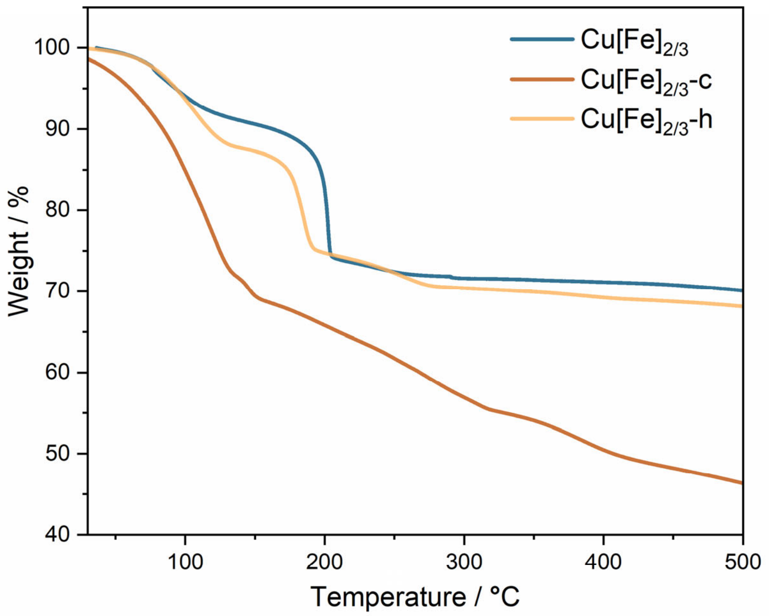

Supplementary Figure 13. TGA profiles of Cu[Fe]$_{2/3}$, Cu[Fe]$_{2/3}$-c, and Cu[Fe]$_{2/3}$-h. Note that Cu[Fe]$_{2/3}$-h is prepared by heating Cu[Fe]$_{2/3}$ at 80 °C and 85 RH% for 72 h, confirming that the increase in interstitial water content is exclusive to Cu[Fe]$_{2/3}$-c. Source data are provided as a Source Data file.

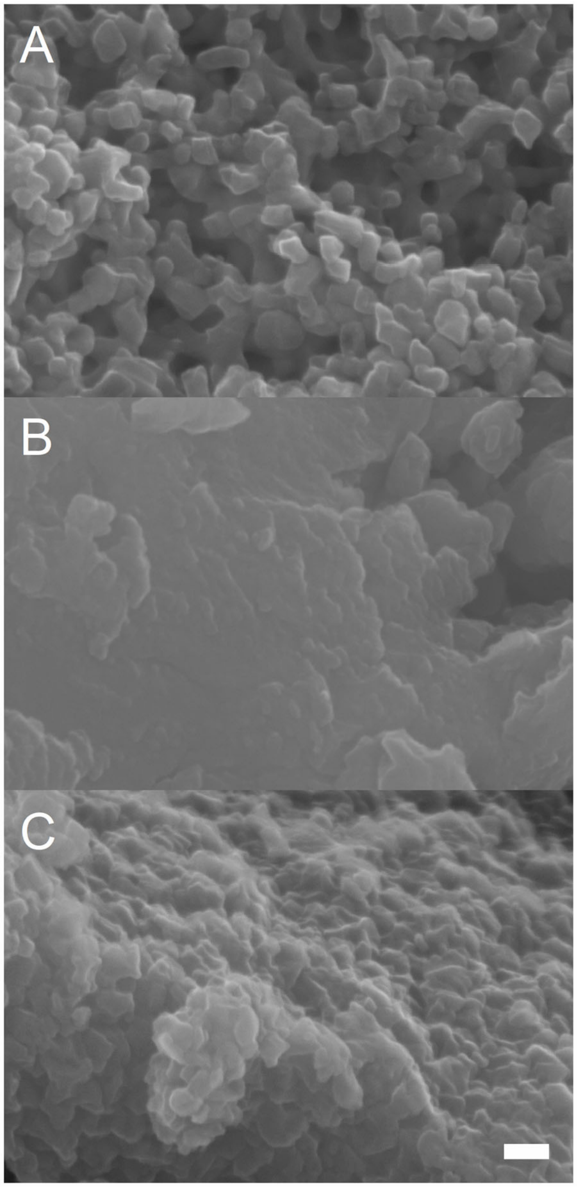

Supplementary Figure 14. SEM images at x100k magnification of (A) $Cu[Fe]_{2/3}$, (B) $Cu[Fe]_{2/3}$-g, and (C) $Cu[Fe]_{2/3}$-c. Scale bar is 100 nm.

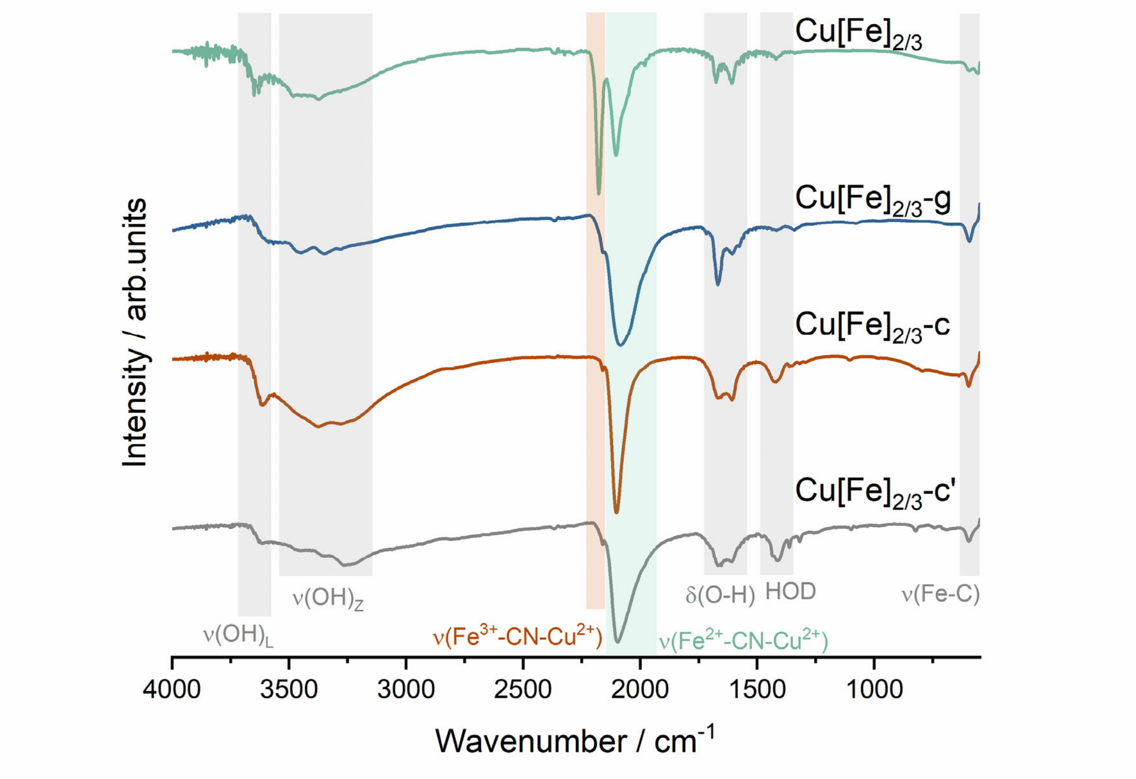

Supplementary Figure 15. FTIR spectra of $Cu[Fe]_{2/3}$, $Cu[Fe]_{2/3}$-g, $Cu[Fe]_{2/3}$-c, and $Cu[Fe]_{2/3}$-c'. Source data are provided as a Source Data file.

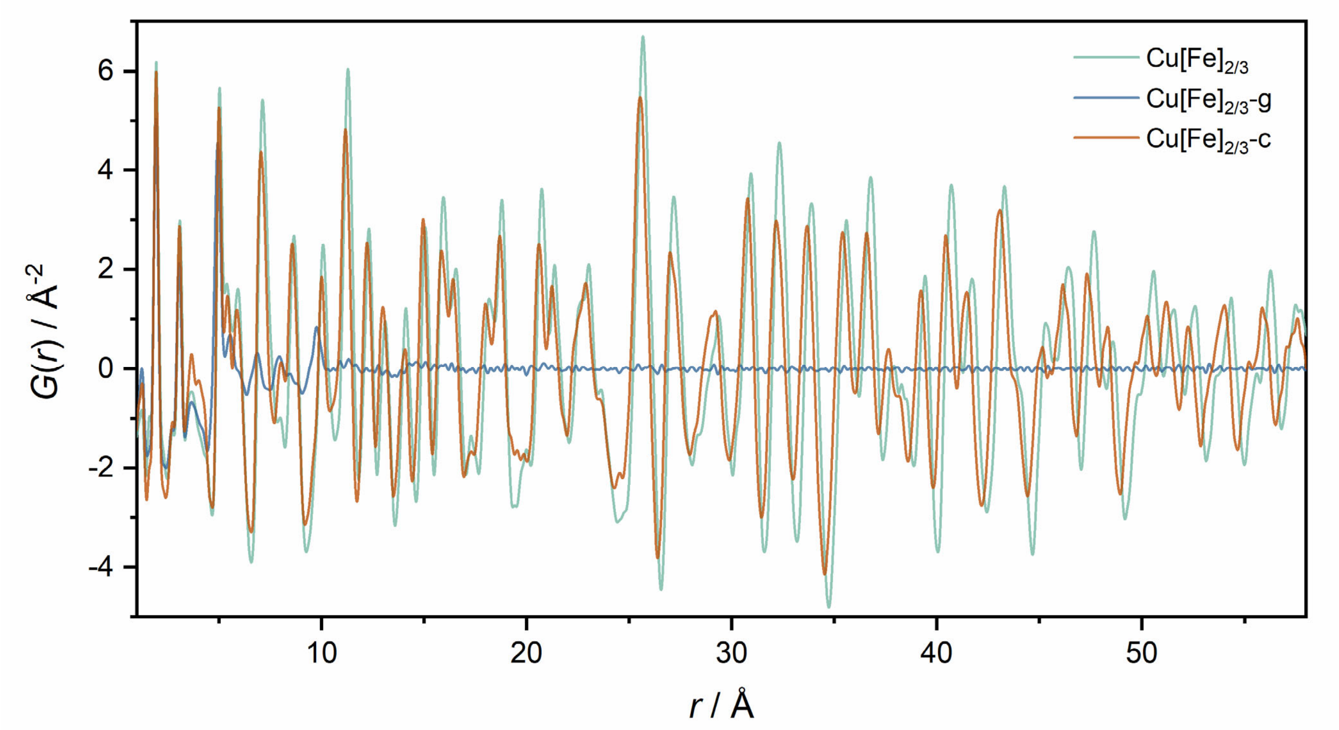

Supplementary Figure 16. Pair distribution function (PDF) of $Cu[Fe]_{2/3}$, $Cu[Fe]_{2/3}$-g, and $Cu[Fe]_{2/3}$-c up to r of 58 Å. Source data are provided as a Source Data file.

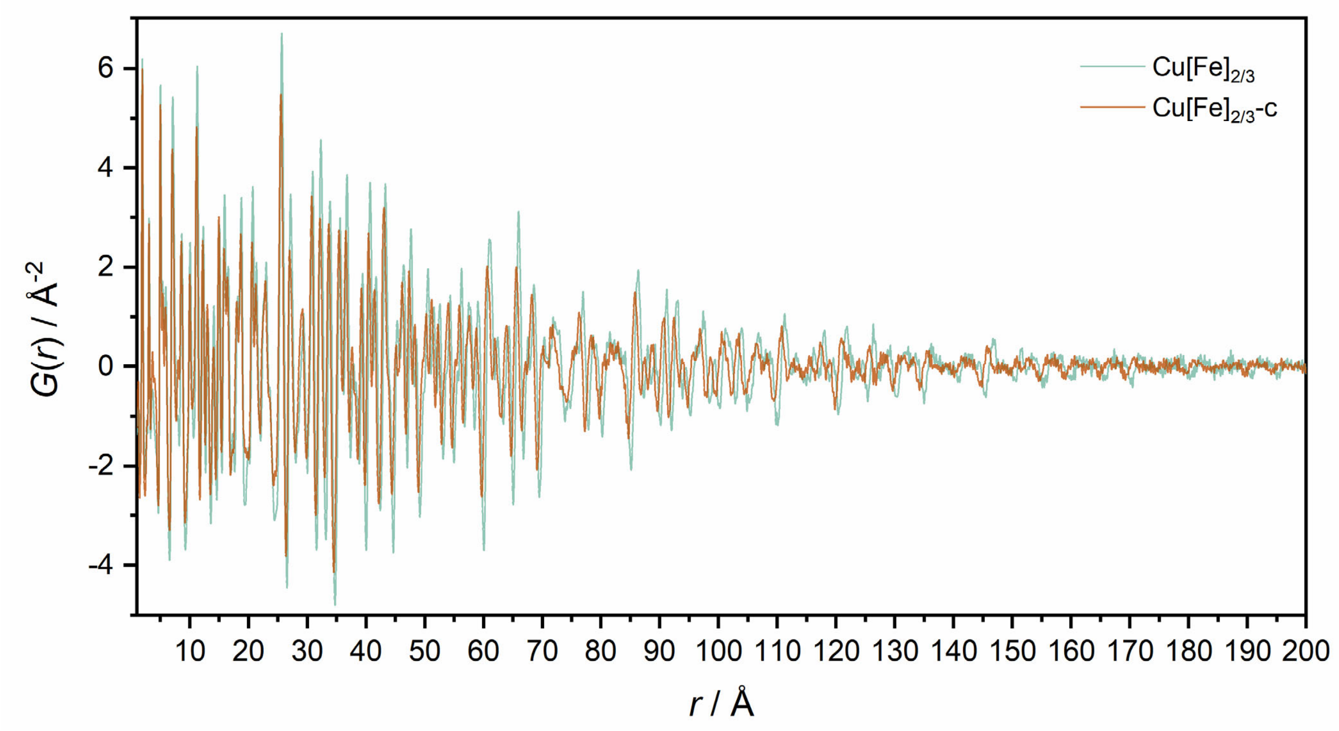

Supplementary Figure 17. Pair distribution function (PDF) of $Cu[Fe]_{2/3}$ and $Cu[Fe]_{2/3}$-c up to *r* of 150 Å. Source data are provided as a Source Data file.

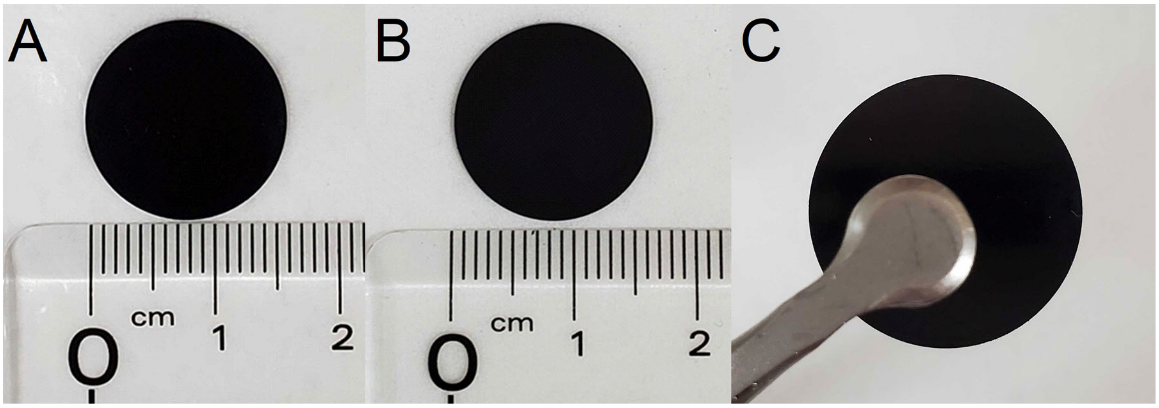

Supplementary Figure 18. Photographs of (A) $Cu[Fe]_{2/3}$ and (B and C) $Cu[Fe]_{2/3}$-g thin monolith disks with a thickness of ca. 250 µm and 16 mm in diameter. The monoliths were prepared via the hot-pressing technique.

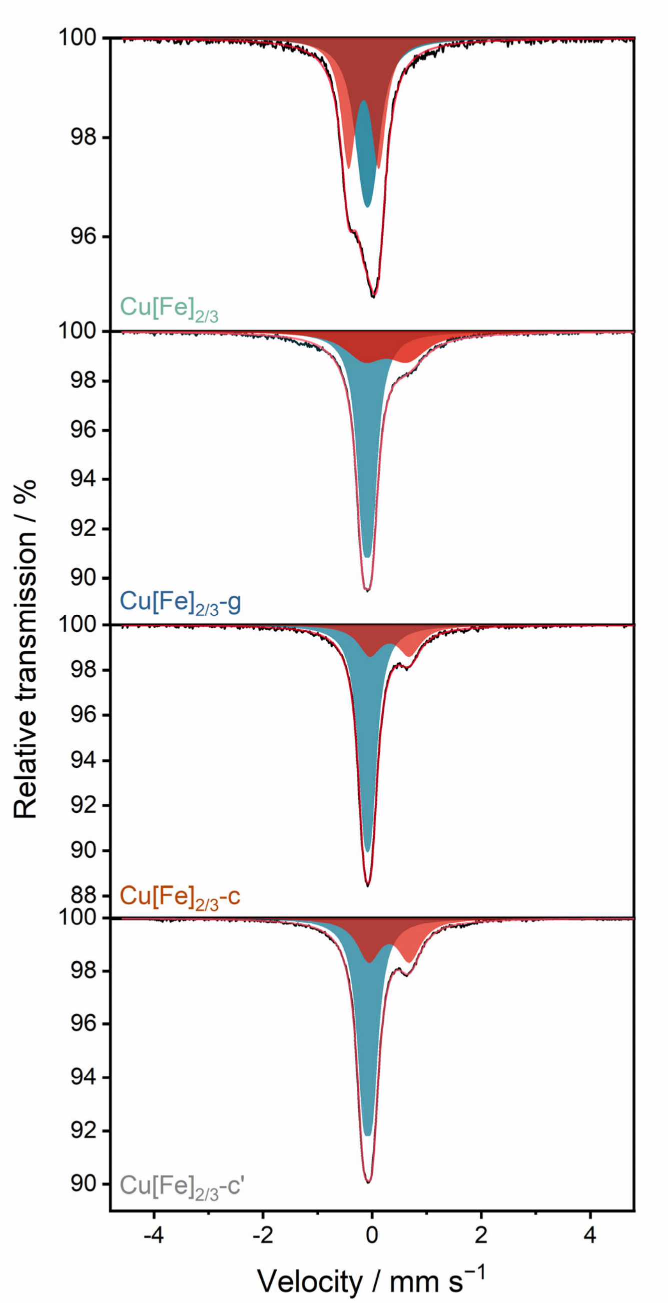

Supplementary Figure 19. ^{57}Fe Mössbauer spectra measured at room temperature of Cu[Fe]$_{2/3}$, Cu[Fe]$_{2/3}$-g, Cu[Fe]$_{2/3}$-c, and Cu[Fe]$_{2/3}$-c'. The blue and red fittings represent the contributions from Fe^{II} and Fe^{III}, respectively. Source data are provided as a Source Data file.

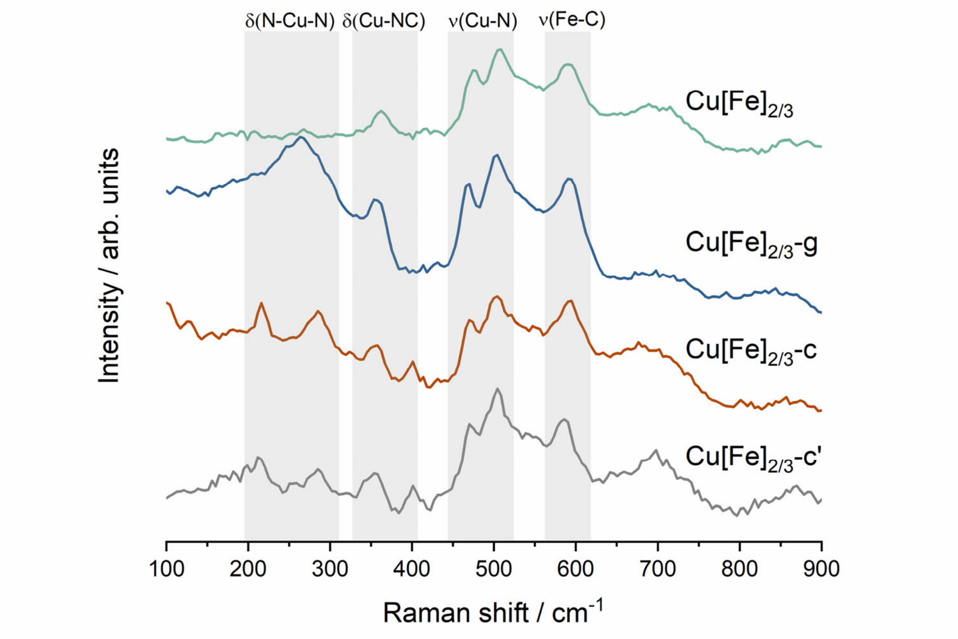

Supplementary Figure 20. Raman spectra of Cu[Fe]$_{2/3}$, Cu[Fe]$_{2/3}$-g, Cu[Fe]$_{2/3}$-c, and Cu[Fe]$_{2/3}$-c'. Source data are provided as a Source Data file.

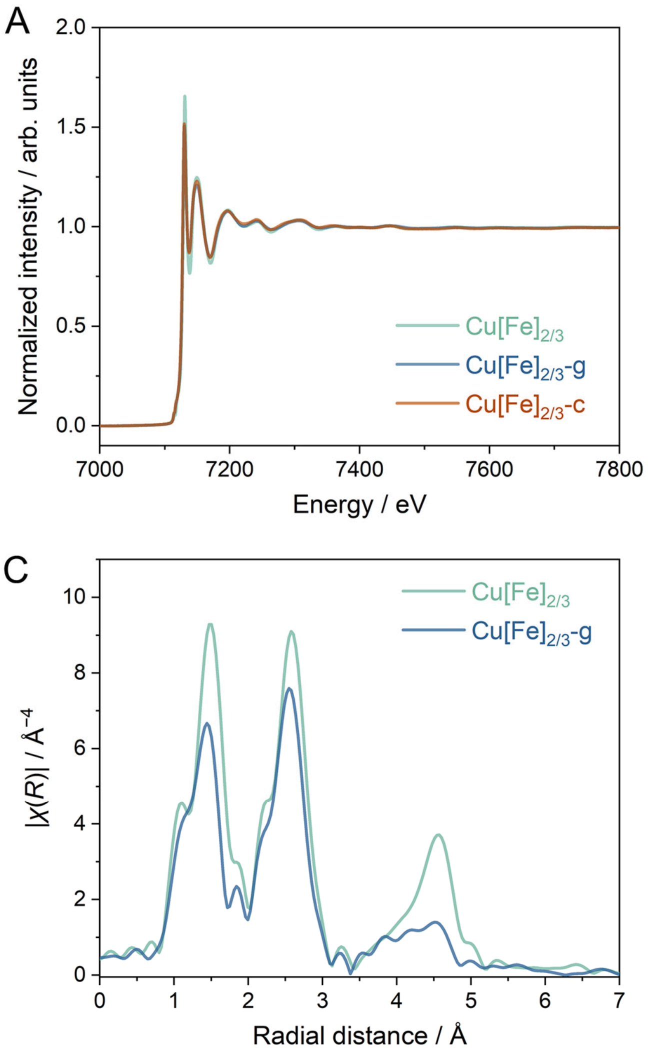

Supplementary Figure 21. (A) Normalised XAS spectra and (B) Fourier transform EXAFS at Fe K-edge of $Cu[Fe]_{2/3}$, $Cu[Fe]_{2/3}$-g, and $Cu[Fe]_{2/3}$-c. Fourier transform EXAFS at Fe K-edge comparison between (C) $Cu[Fe]_{2/3}$ – $Cu[Fe]_{2/3}$-g and (D) $Cu[Fe]_{2/3}$-g – $Cu[Fe]_{2/3}$-c. Source data are provided as a Source Data file.

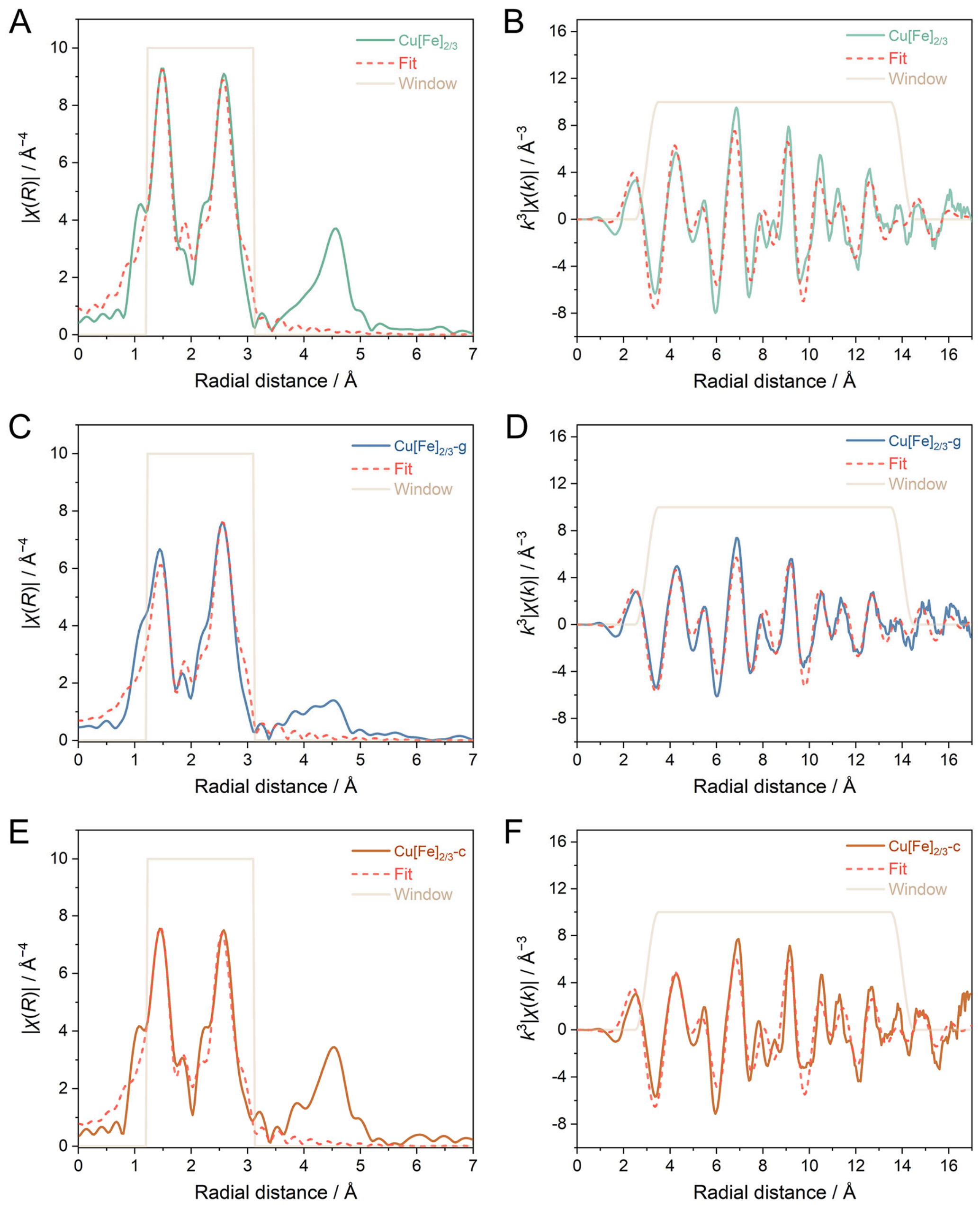

Supplementary Figure 22. Fourier transform and EXAFS functions at Fe K-edge for (A, B) Cu[Fe]$_{2/3}$, (C, D) Cu[Fe]$_{2/3}$-g, and (E, F) Cu[Fe]$_{2/3}$-c. The fitting results show that the coordination numbers of Fe are 6.0, 4.4, and 5.4 for Cu[Fe]$_{2/3}$, Cu[Fe]$_{2/3}$-g, and Cu[Fe]$_{2/3}$-c, respectively (Table S4). Source data are provided as a Source Data file.

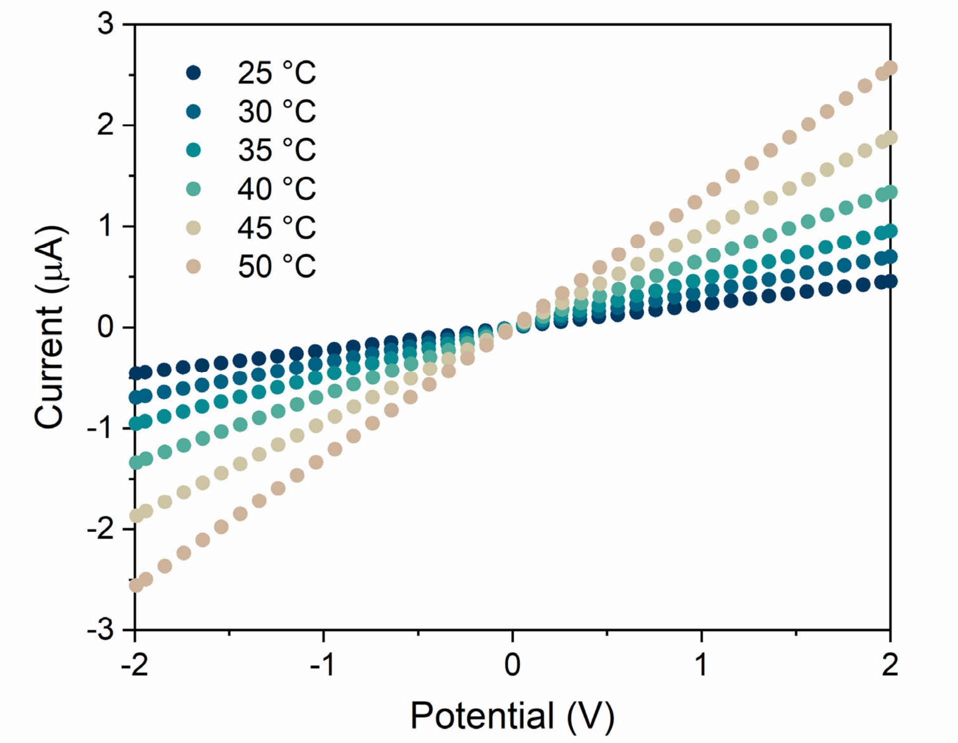

Supplementary Figure 23. Variable temperature I-V curve of Cu[Fe]$_{2/3}$ (L = 1.524 mm). Source data are provided as a Source Data file.

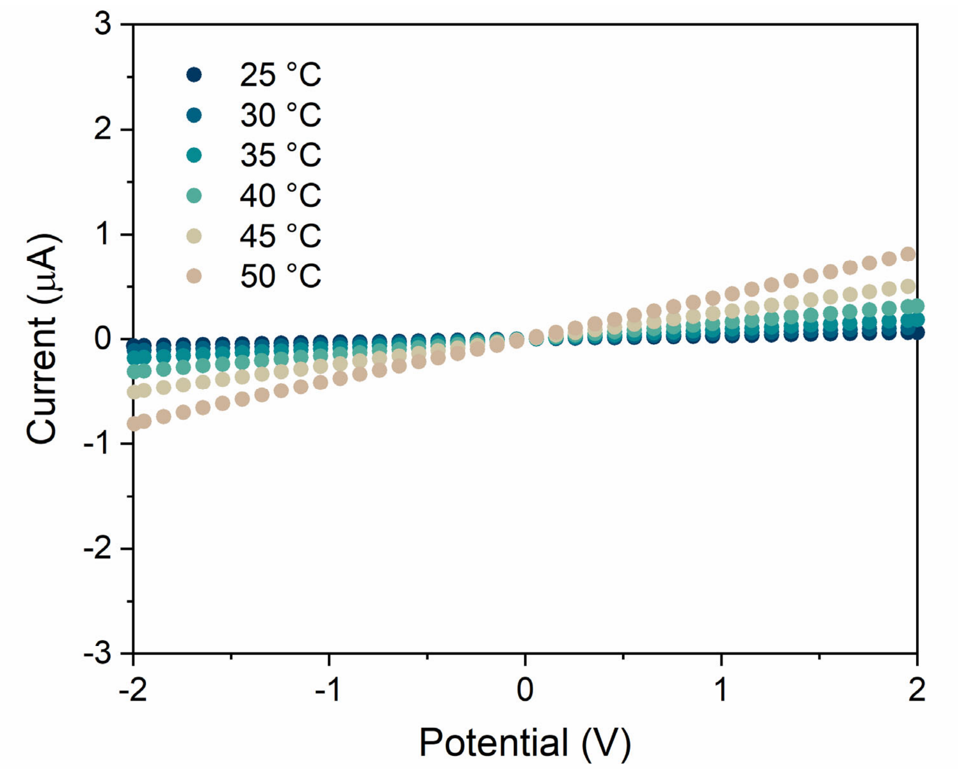

Supplementary Figure 24. Variable temperature I-V curve of Cu[Fe]$_{2/3}$-g (L = 1.469 mm). Source data are provided as a Source Data file.

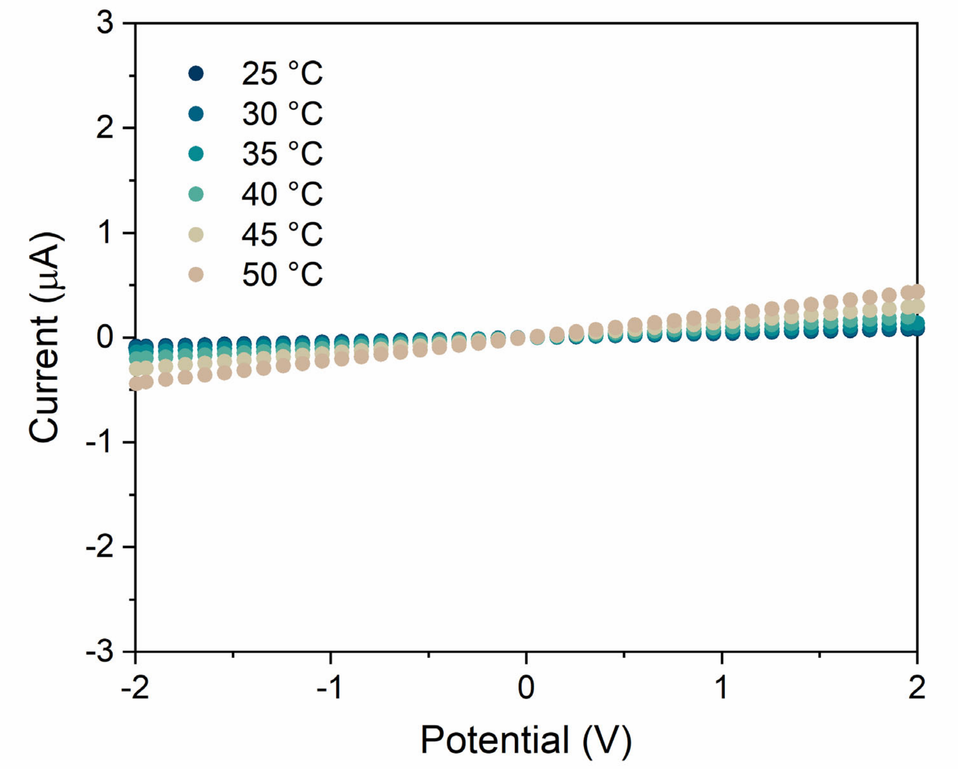

Supplementary Figure 25. Variable temperature I-V curve of Cu[Fe]$_{2/3}$-c' (L = 1.506 mm). Source data are provided as a Source Data file.

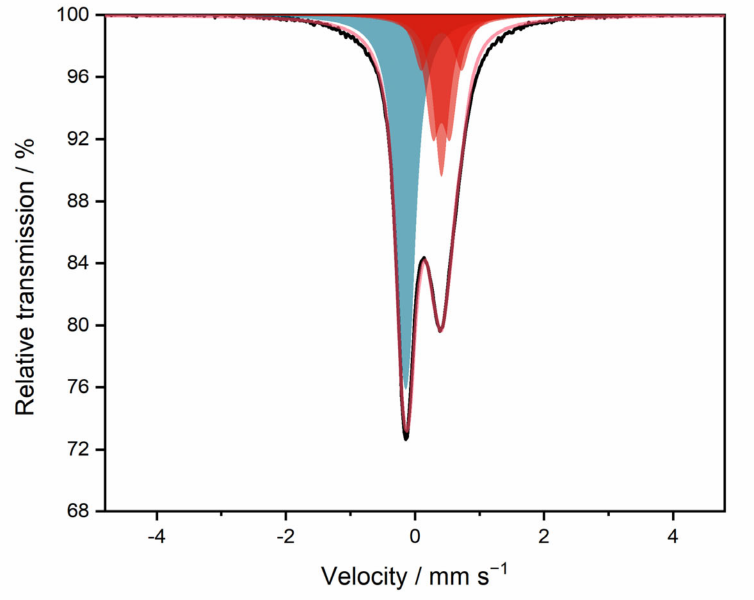

Supplementary Figure 26. ^{57}Fe Mössbauer spectra measured at room temperature of Fe[Fe]3/4. The blue and red fittings represent the contributions from low-spin Fe^{II} and high-spin Fe^{III}, respectively. The spectrum was analysed in terms of a single low-spin Fe^{II} quadrupole doublet and three high-spin Fe^{III} quadrupole doublets.[1] Source data are provided as a Source Data file.

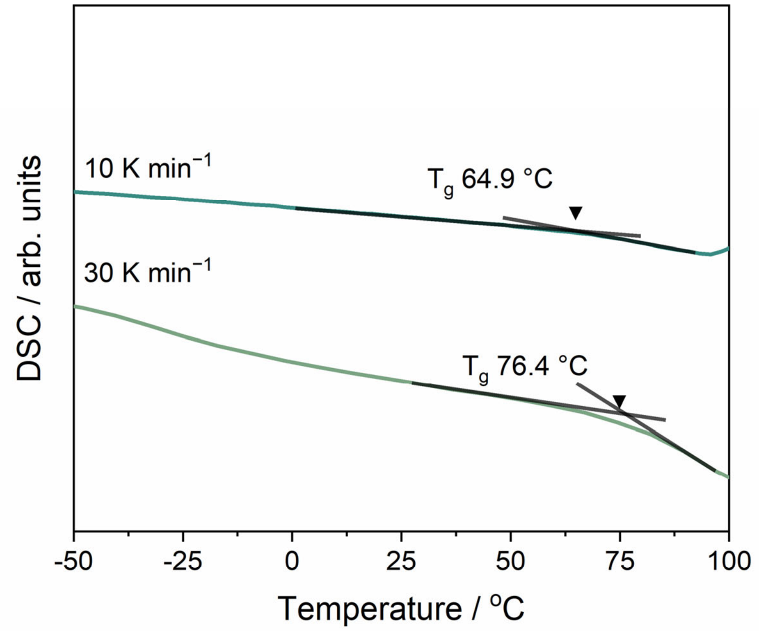

Supplementary Figure 27. DSC profile of Fe[Fe]3/4-g after 144 h of mechanical milling. Source data are provided as a Source Data file.

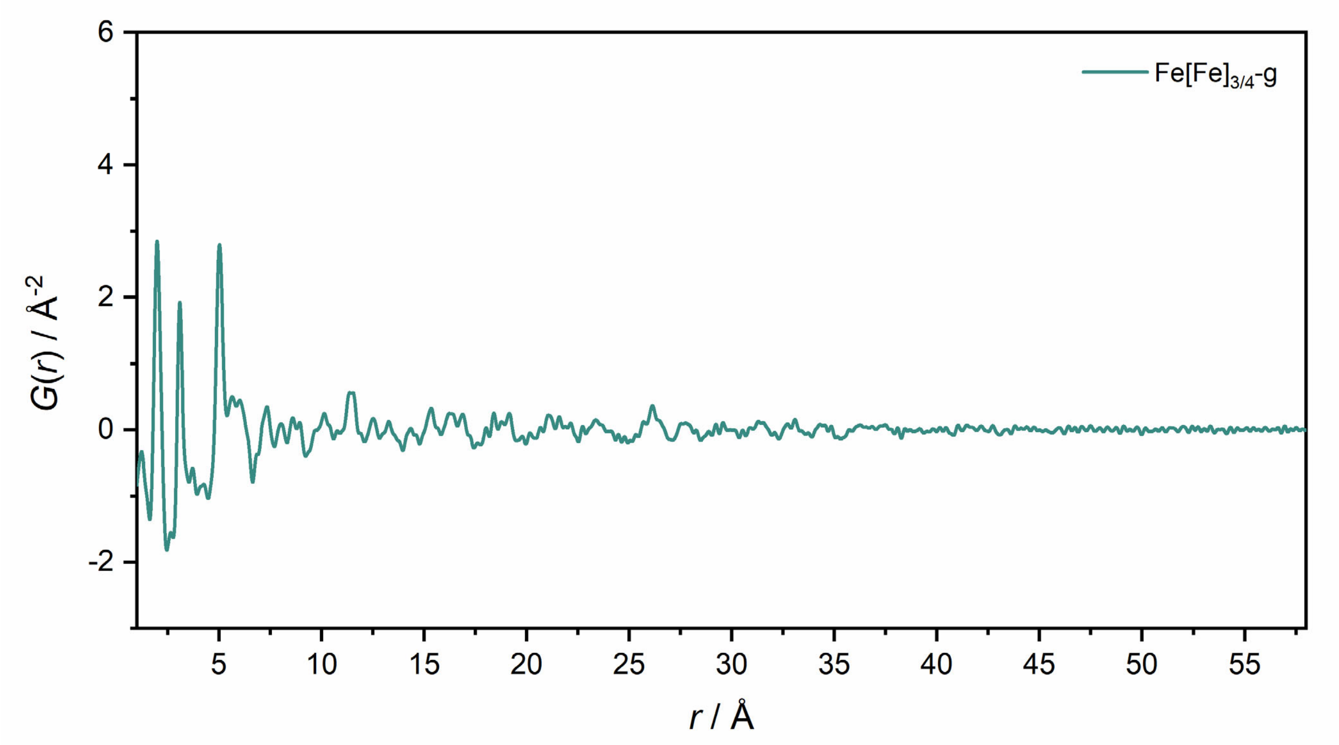

Supplementary Figure 28. Pair distribution function (PDF) of $Fe[Fe]_{3/4}$-g up to *r* of 58 Å. Source data are provided as a Source Data file.

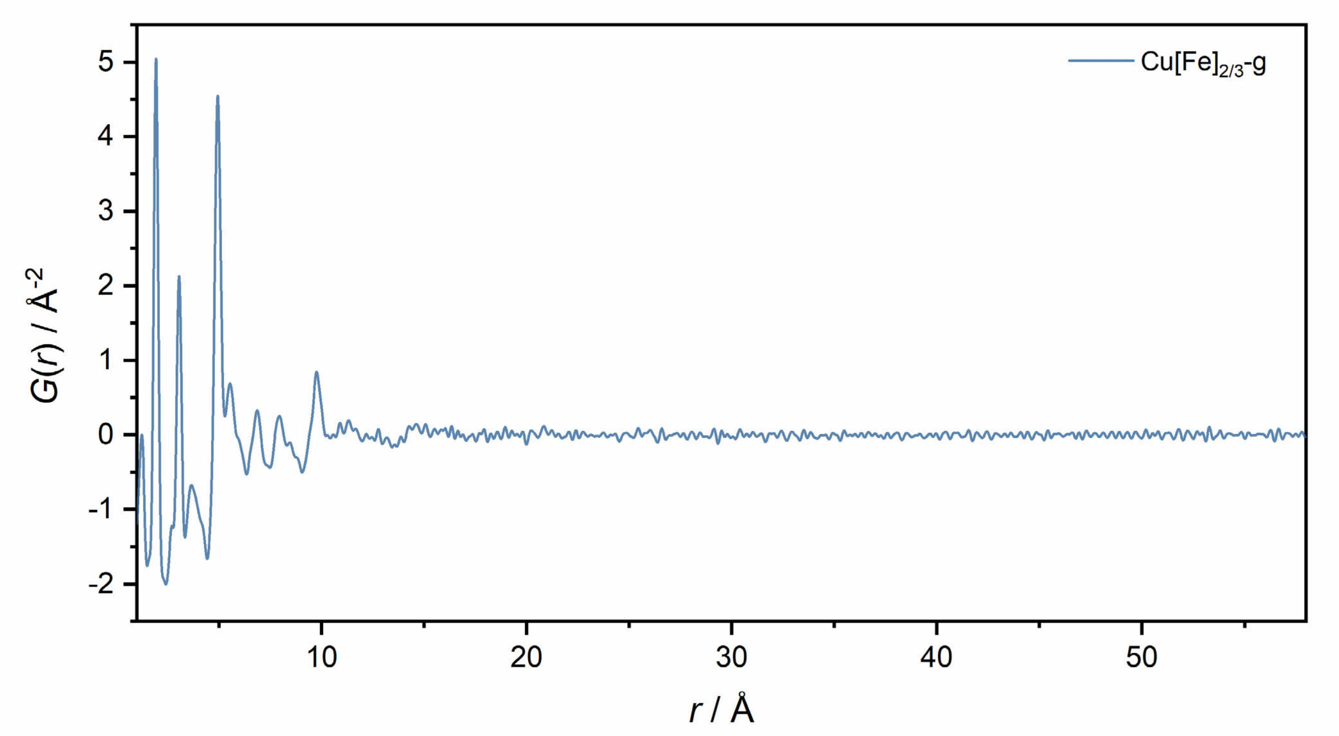

Supplementary Figure 29. Pair distribution function (PDF) of $Cu[Fe]_{2/3}$-g up to *r* of 58 Å. Source data are provided as a Source Data file.

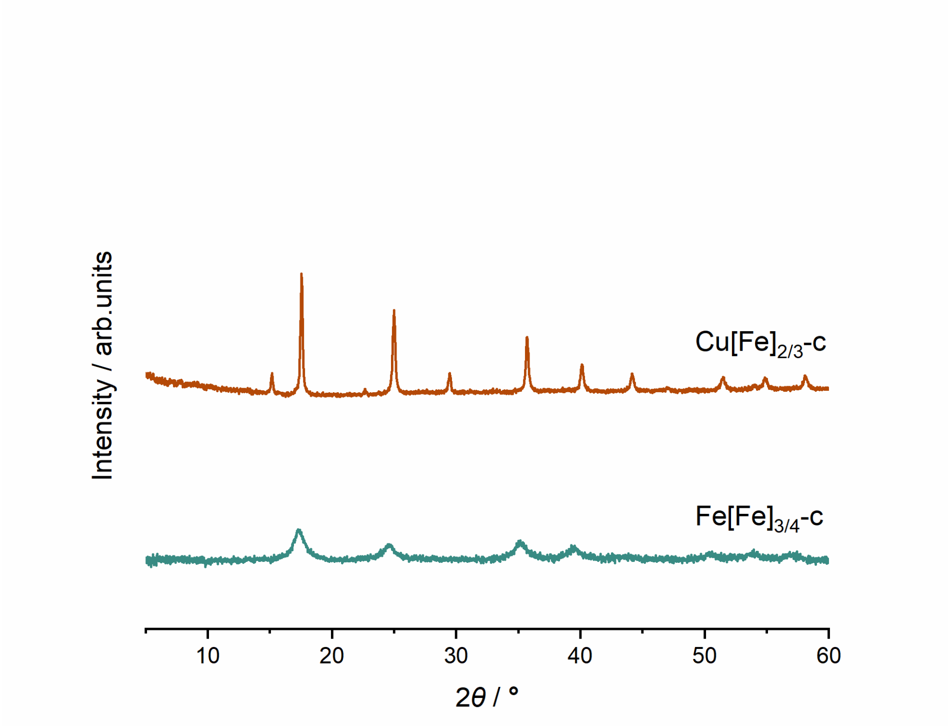

Supplementary Figure 30. PXRD patterns of Cu[Fe]$_{2/3}$-c and Fe[Fe]$_{3/4}$-c (λ = 1.5406 Å). Source data are provided as a Source Data file.

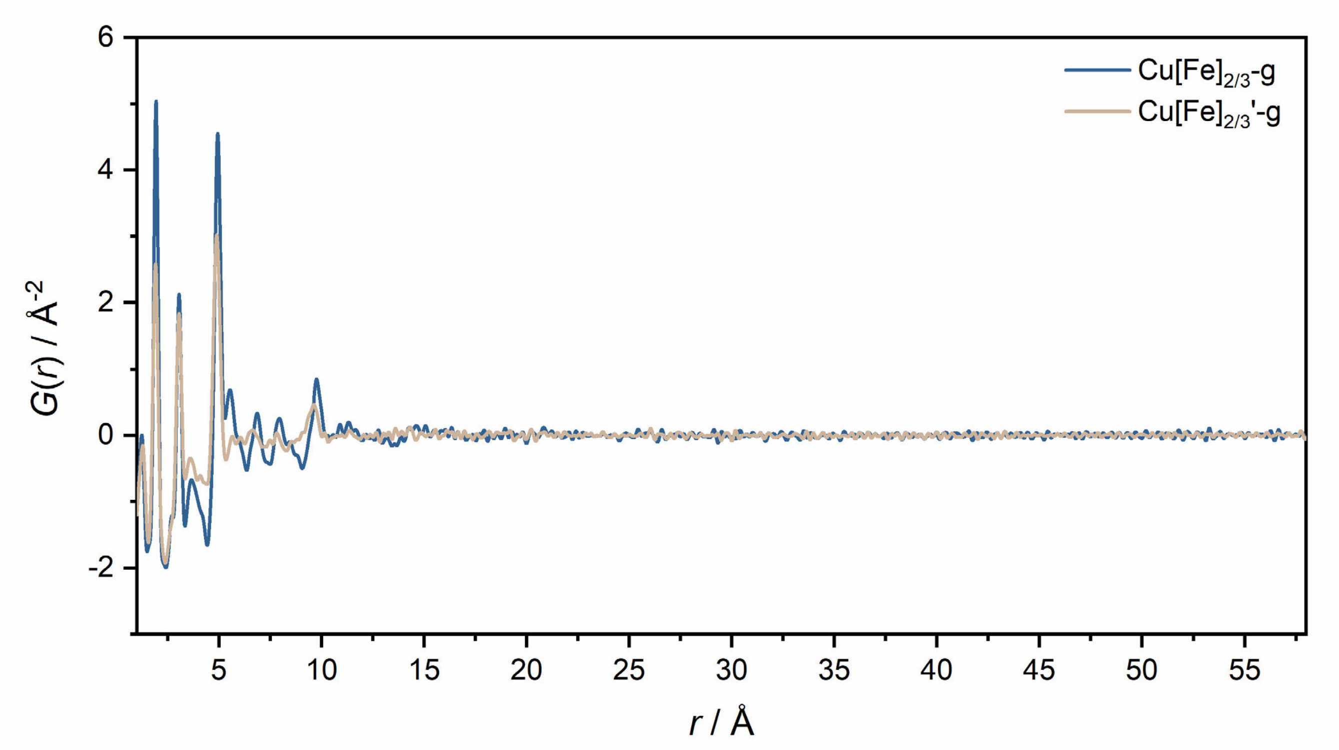

Supplementary Figure 31. Pair distribution functions (PDFs) of Cu[Fe]$_{2/3}$-g and Cu[Fe]$_{2/3}$'-g up to *r* of 58 Å. Source data are provided as a Source Data file.

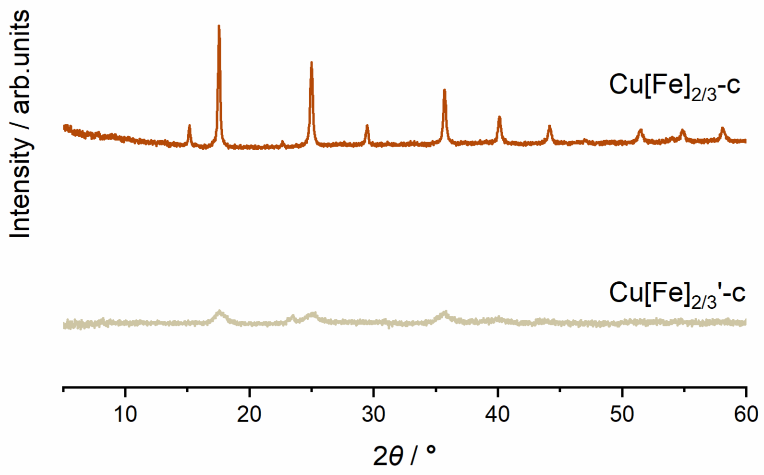

Supplementary Figure 32. PXRD patterns of $Cu[Fe]_{2/3}$-c and $Cu[Fe]_{2/3}$'-c (λ = 1.5406 Å). Source data are provided as a Source Data file.

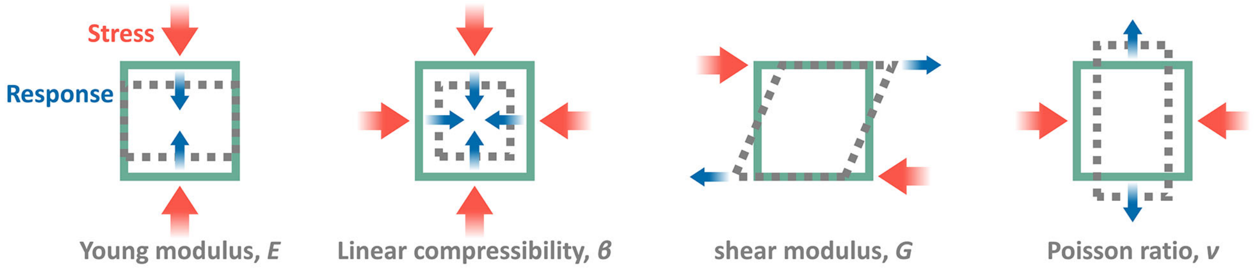

Supplementary Figure 33. Schematic illustration of the directional elastic properties of materials in 2D space.

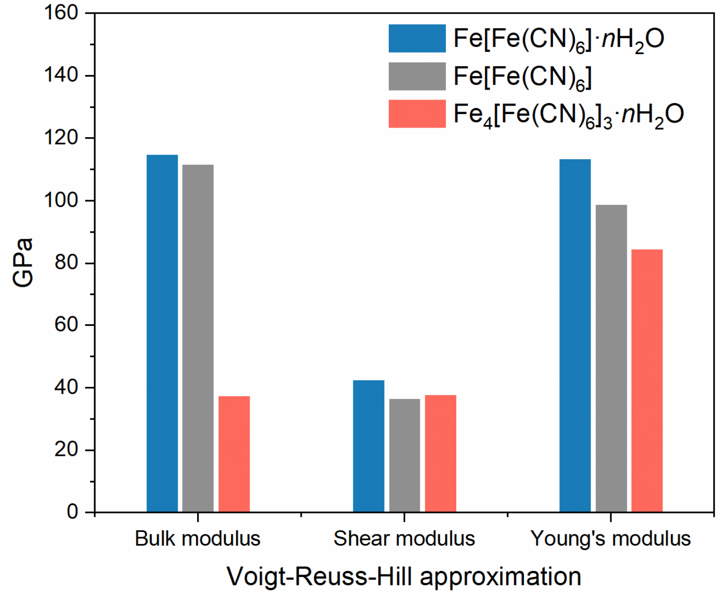

Supplementary Figure 34. Bulk, shear, and Young's moduli comparison of $Fe[Fe(CN)_6]\cdot nH_2O$, $Fe[Fe(CN)_6]$, and $Fe_4[Fe(CN)_6]_3\cdot nH_2O$ at 0 GPa by Voigt-Reuss-Hill approximation. Source data are provided as a Source Data file.

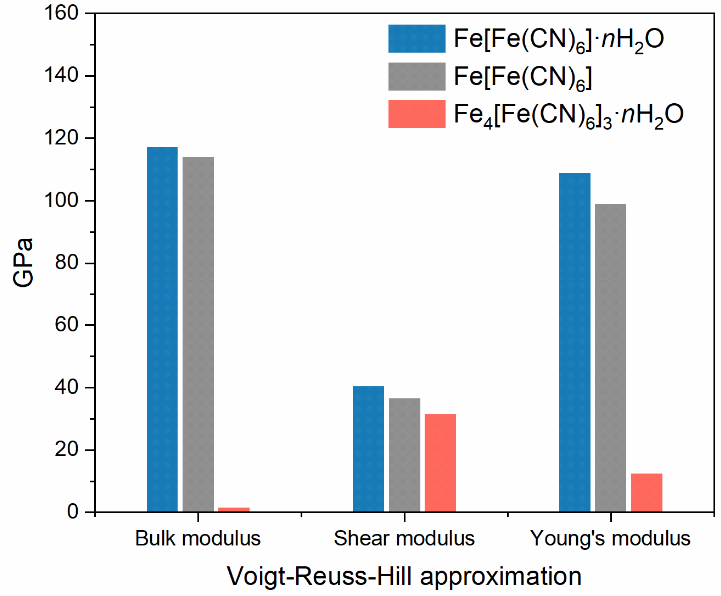

Supplementary Figure 35. Bulk, shear, and Young's moduli comparison of $Fe[Fe(CN)_6]\cdot nH_2O$, $Fe[Fe(CN)_6]$, and $Fe_4[Fe(CN)_6]_3\cdot nH_2O$ at 0.2 GPa by Voigt-Reuss-Hill approximation. Note, the $Fe_4[Fe(CN)_6]_3\cdot nH_2O$ is considered unstable according to the Eigenvalue analysis. Source data are provided as a Source Data file.

Spatial elastic

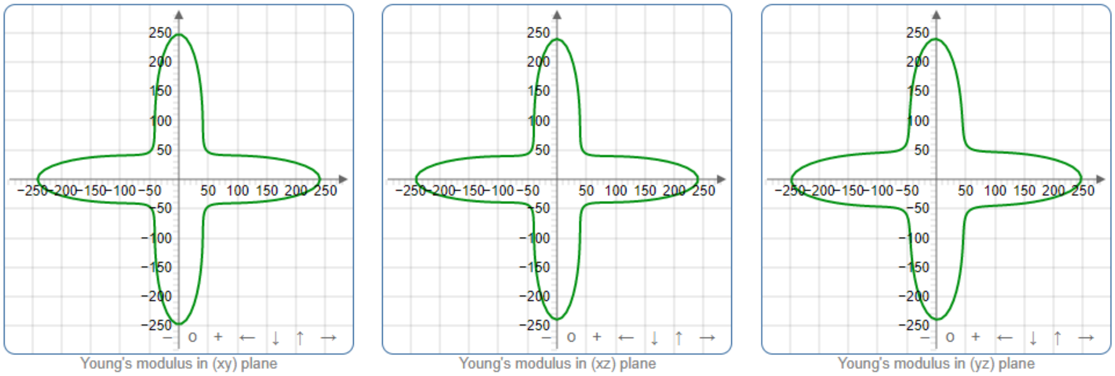

Supplementary Figure 36. Spatial dependence Young's modulus of the $Fe[Fe(CN)_6]\cdot nH_2O$ at 0 GPa. Source data are provided as a Source Data file.

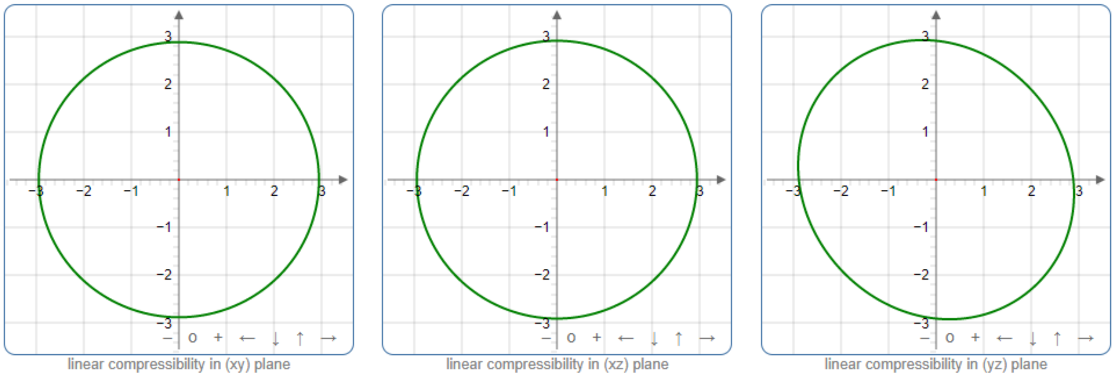

Supplementary Figure 37. Spatial dependence linear compressibility of the $Fe[Fe(CN)_6]\cdot nH_2O$ at 0 GPa. Source data are provided as a Source Data file.

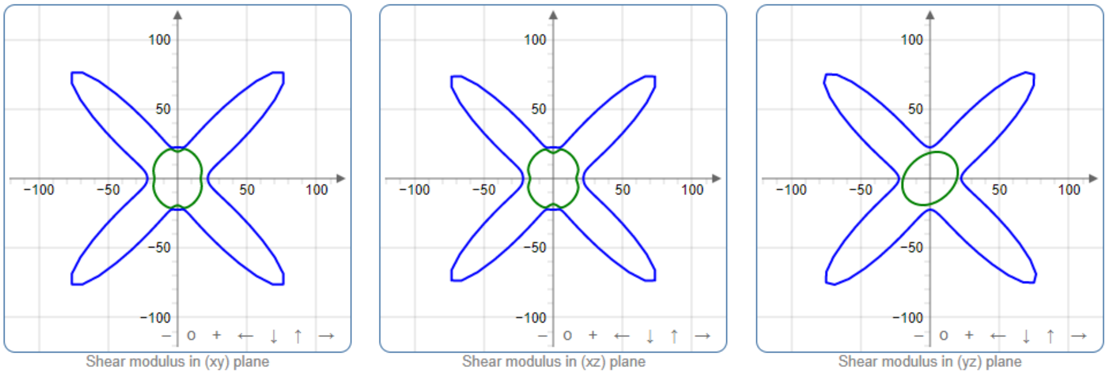

Supplementary Figure 38. Spatial dependence shear modulus of the $Fe[Fe(CN)_6]\cdot nH_2O$ at 0 GPa. Blue and green curves/surfaces represent maximum and positive minimum shear moduli, respectively. Source data are provided as a Source Data file.

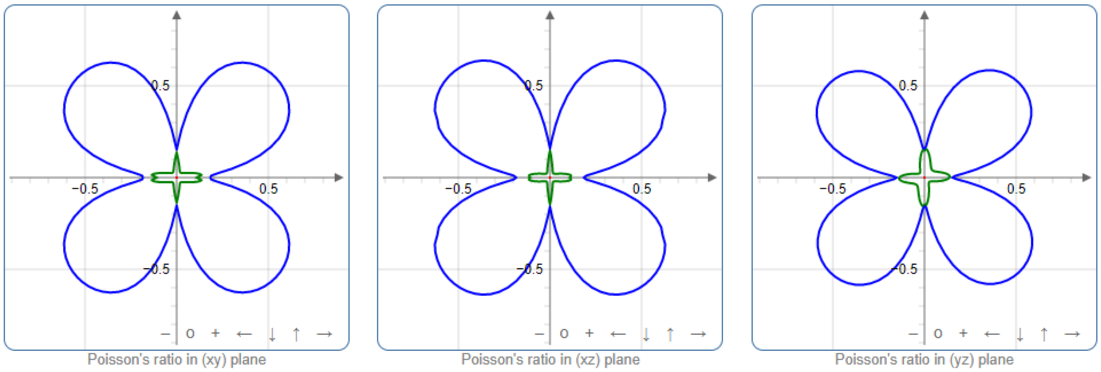

Supplementary Figure 39. Spatial dependence Poisson's ratio of the Fe[Fe(CN)$_6$]$\cdot n$H$_2$O at 0 GPa. Blue, green, and red curves/surfaces represent maximum, positive minimum, and negative minimum Poisson's ratios, respectively. Source data are provided as a Source Data file.

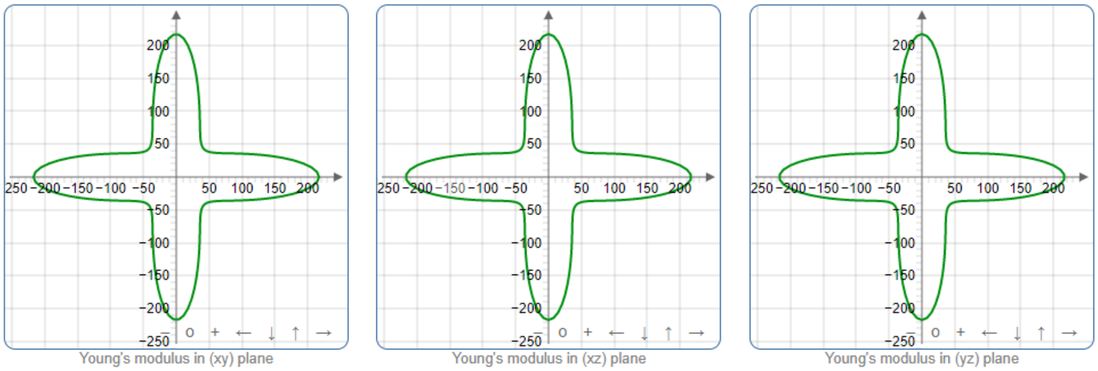

Supplementary Figure 40. Spatial dependence Young's modulus of the Fe[Fe(CN)$_6$] at 0 GPa. Source data are provided as a Source Data file.

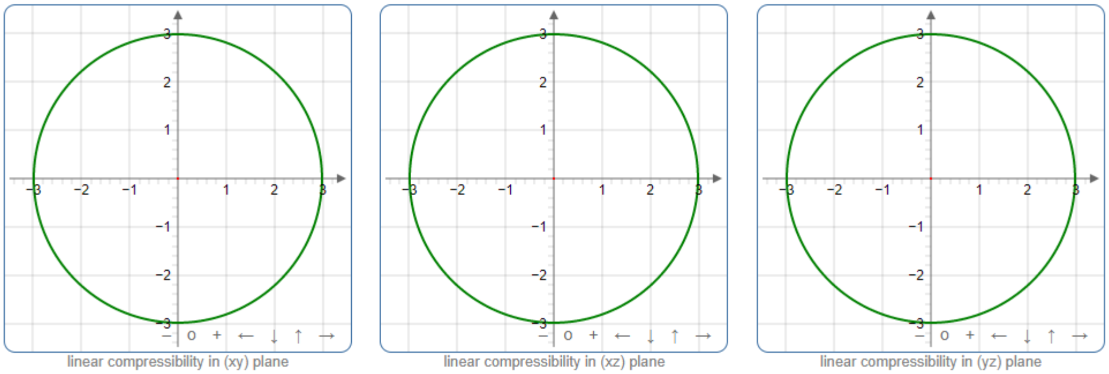

Supplementary Figure 41. Spatial dependence linear compressibility of the Fe[Fe(CN)$_6$] at 0 GPa. Source data are provided as a Source Data file.

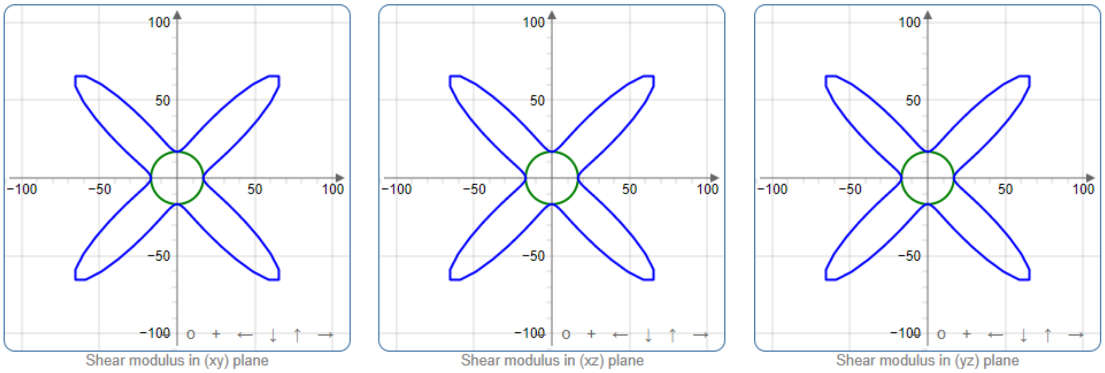

Supplementary Figure 42. Spatial dependence shear modulus of the Fe[Fe(CN)$_6$] at 0 GPa. Blue and green curves/surfaces represent maximum and positive minimum shear moduli, respectively. Source data are provided as a Source Data file.

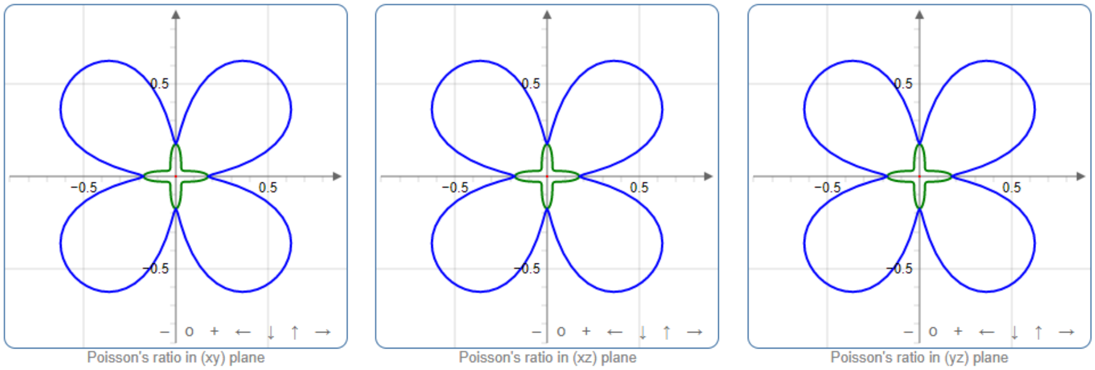

Supplementary Figure 43. Spatial dependence Poisson's ratio of the Fe[Fe(CN)$_6$] at 0 GPa. Blue, green, and red curves/surfaces represent maximum, positive minimum, and negative minimum Poisson's ratios, respectively. Source data are provided as a Source Data file.

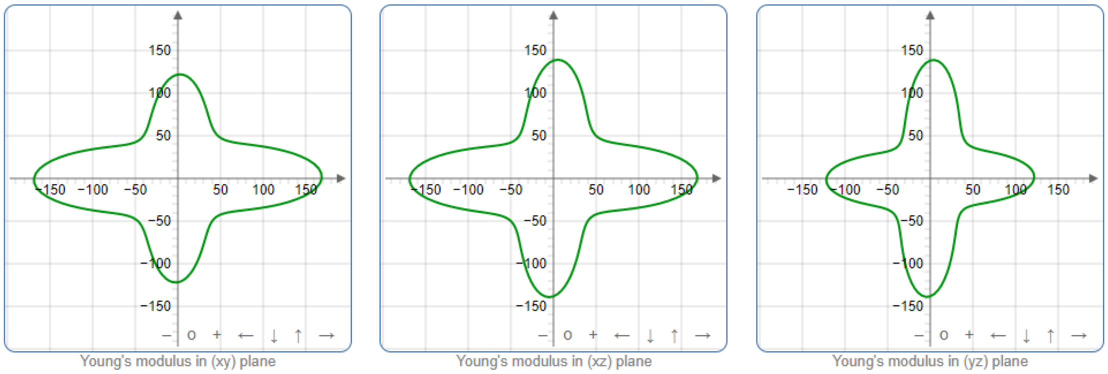

Supplementary Figure 44. Spatial dependence Young's modulus of the $Fe_4[Fe(CN)_6]_3 \cdot nH_2O$ at 0 GPa. Source data are provided as a Source Data file.

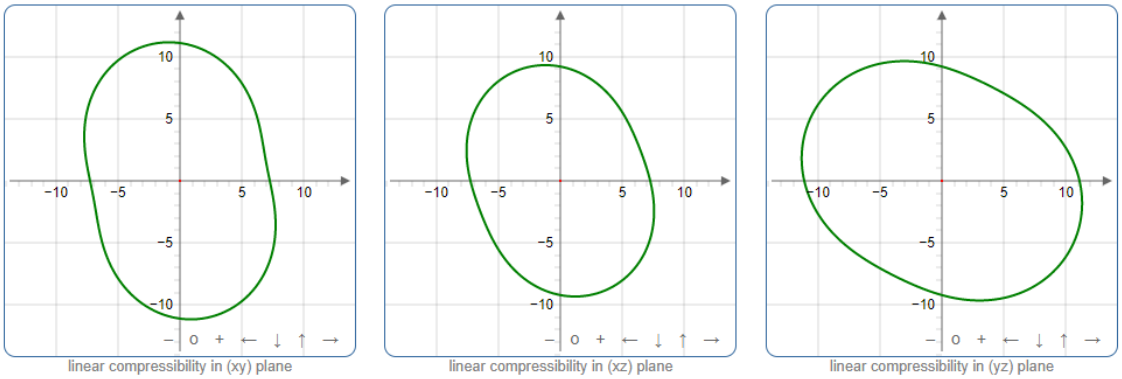

Supplementary Figure 45. Spatial dependence linear compressibility of the $Fe_4[Fe(CN)_6]_3{\cdot}nH_2O$ at 0 GPa. Source data are provided as a Source Data file.

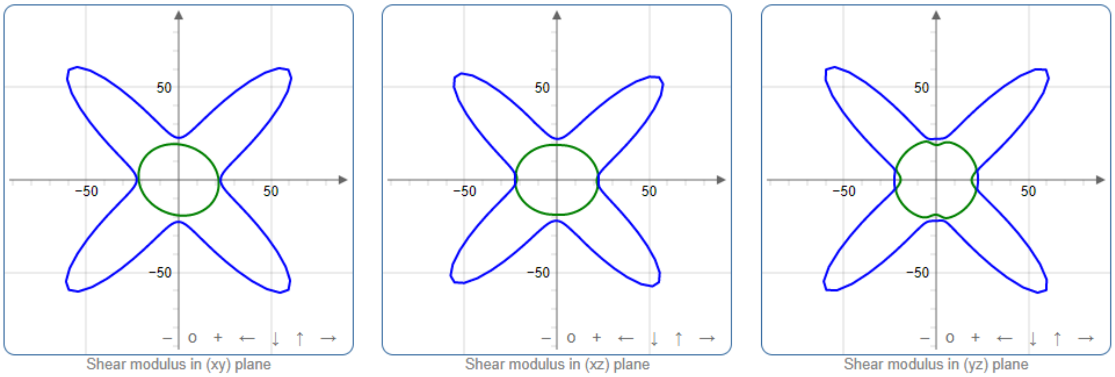

Supplementary Figure 46. Spatial dependence shear modulus of the $Fe_4[Fe(CN)_6]_3{\cdot}nH_2O$ at 0 GPa. Blue and green curves/surfaces represent maximum and positive minimum shear moduli, respectively. Source data are provided as a Source Data file.

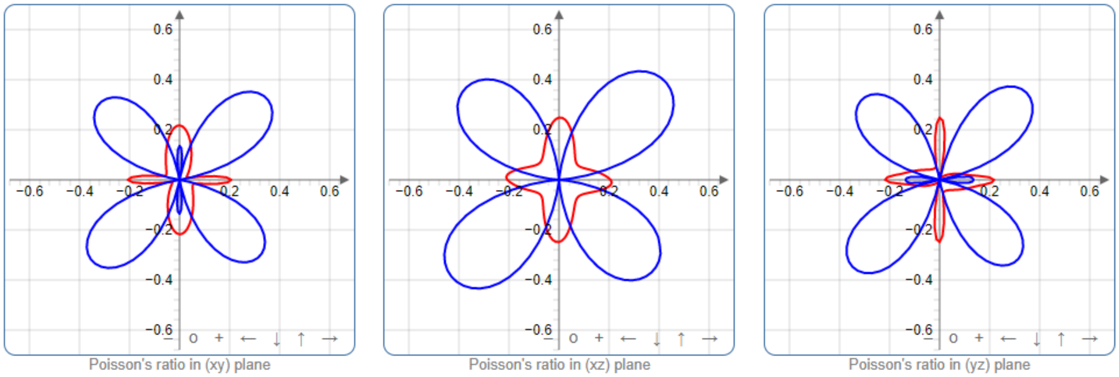

Supplementary Figure 47. Spatial dependence Poisson's ratio of the $Fe_4[Fe(CN)_6]_3{\cdot}nH_2O$ at 0 GPa. Blue, green, and red curves/surfaces represent maximum, positive minimum, and negative minimum Poisson's ratios, respectively. Source data are provided as a Source Data file.

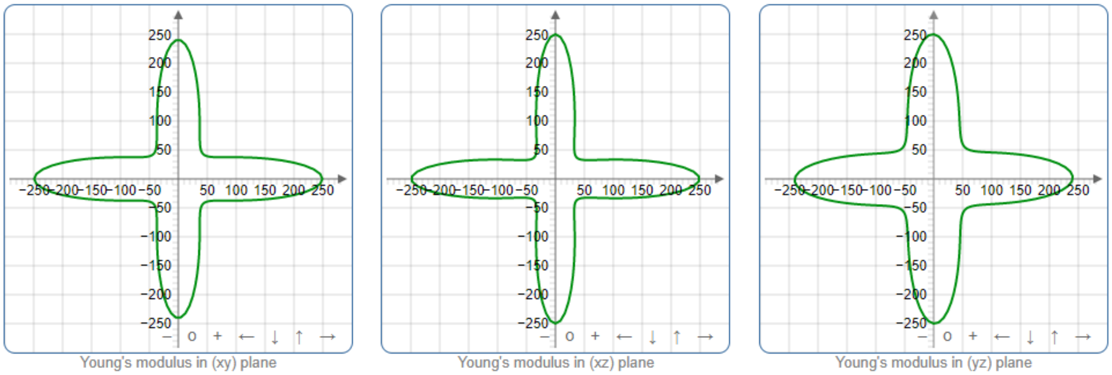

Supplementary Figure 48. Spatial dependence Young's modulus of the $Fe[Fe(CN)_6]\cdot nH_2O$ at 0.2 GPa. Source data are provided as a Source Data file.

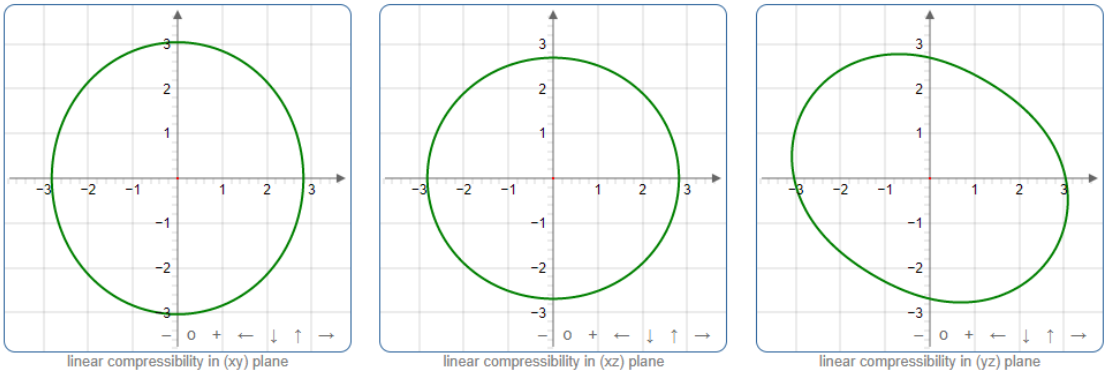

Supplementary Figure 49. Spatial dependence linear compressibility of the $Fe[Fe(CN)_6]\cdot nH_2O$ at 0.2 GPa. Source data are provided as a Source Data file.

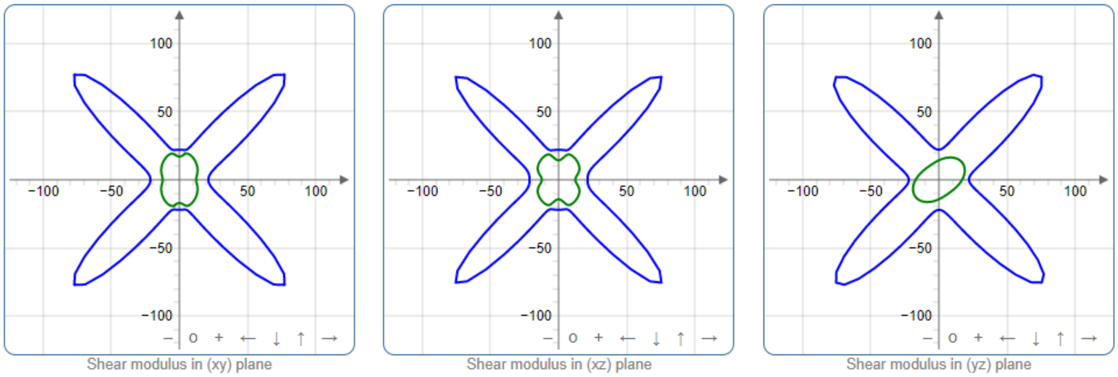

Supplementary Figure 50. Spatial dependence shear modulus of the $Fe[Fe(CN)_6]\cdot nH_2O$ at 0.2 GPa. Blue and green curves/surfaces represent maximum and positive minimum shear moduli, respectively. Source data are provided as a Source Data file.

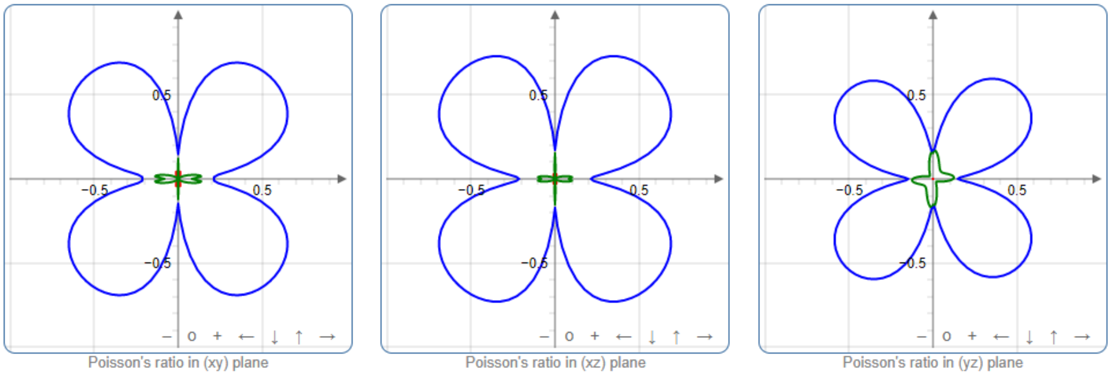

Supplementary Figure 51. Spatial dependence Poisson's ratio of the Fe[Fe(CN)$_6$]·nH$_2$O at 0.2 GPa. Blue, green, and red curves/surfaces represent maximum, positive minimum, and negative minimum Poisson's ratios, respectively. Source data are provided as a Source Data file.

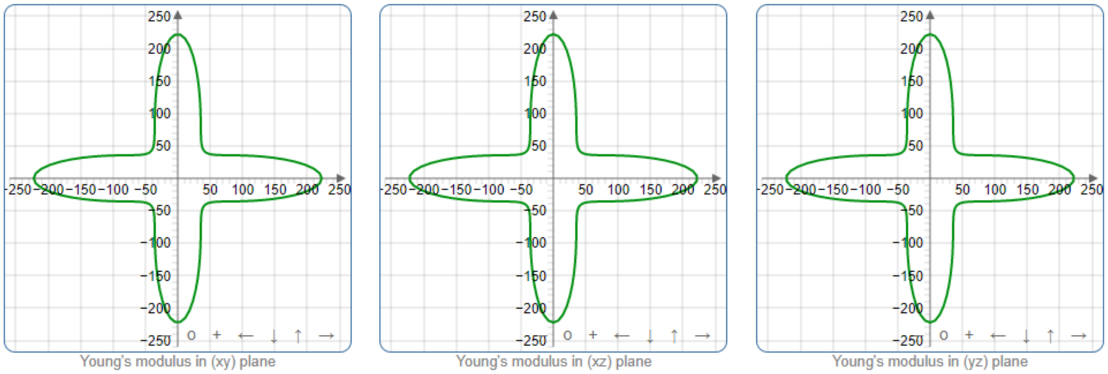

Supplementary Figure 52. Spatial dependence Young's modulus of the Fe[Fe(CN)$_6$] at 0.2 GPa. Source data are provided as a Source Data file.

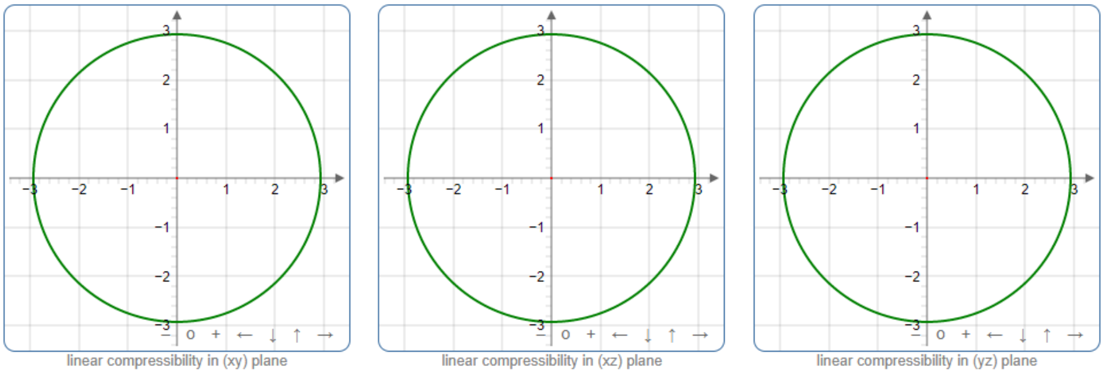

Supplementary Figure 53. Spatial dependence linear compressibility of the Fe[Fe(CN)$_6$] at 0.2 GPa. Source data are provided as a Source Data file.

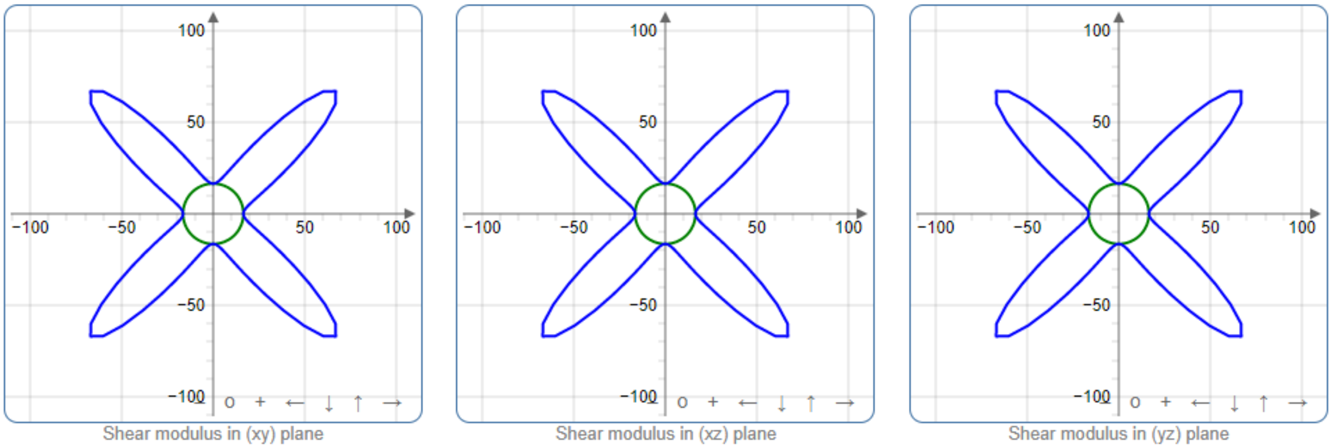

Supplementary Figure 54. Spatial dependence shear modulus of the $Fe[Fe(CN)_6]$ at 0.2 GPa. Blue and green curves/surfaces represent maximum and positive minimum shear moduli, respectively. Source data are provided as a Source Data file.

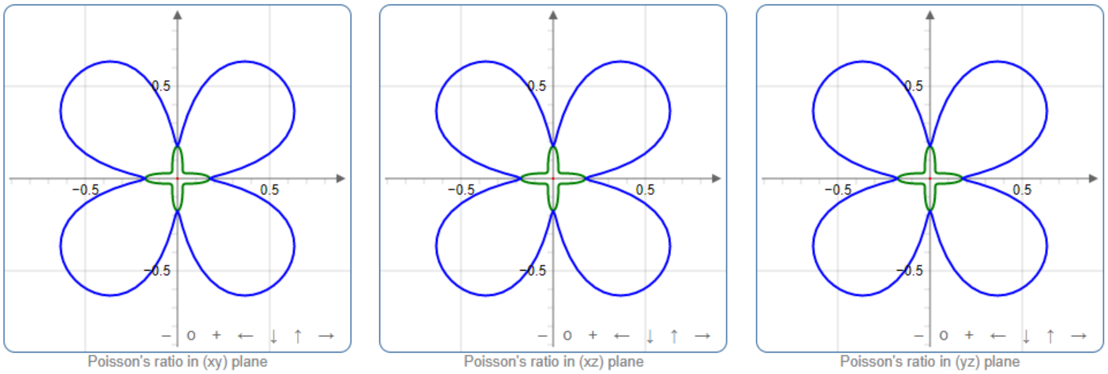

Supplementary Figure 55. Spatial dependence Poisson's ratio of the $Fe[Fe(CN)_6]$ at 0.2 GPa. Blue, green, and red curves/surfaces represent maximum, positive minimum, and negative minimum Poisson's ratios, respectively. Source data are provided as a Source Data file.

Supplementary Reference

1. Grandjean, F., Samain, L. & Long, G.J. Characterization and utilization of Prussian blue and its pigments. *Dalton Trans.* **45**, 18018–18044 (2016).
